# Supplementary material for: Aggressive signaling among competing species of birds
Source: PeerJ. 2022 Jun 13;10:e13431. doi: 10.7717/peerj.13431 (PMC9202552; doi:10.7717/peerj.13431)
Supplement: Supplemental Information 3 [file peerj-10-13431-s003.docx]

**SUPPORTING INFORMATION**

**Table of contents:**

**APPENDIX I**: Posture definitions and examples – page 2

**APPENDIX II**: Instructions for naïve observers examining images for body regions and colors most highlighted in interspecific aggressive signals – page 9

**APPENDIX III**: Color names considered to be synonymous with each focal color or color group – page 10

**APPENDIX IV**: Phylogeny of species included in dataset – page 11

**APPENDIX V**: Posture model results and diagnostics – page 12

**APPENDIX VI**: Posture model results from subset of data obtained from photographs – page 39

**APPENDIX VII**: Highlighted body regions and highlighted colors model results and diagnostics – page 41

**APPENDIX VIII**: Highlighted body regions and colors model results from subset of data obtained from photographs – page 49

**APPENDIX IX**: Degree of similarity to intraspecific aggressive signal model results and diagnostics – page 50

**APPENDIX X:** Degree of similarity to intraspecific aggressive signal model results from subset of data obtained from photographs – page 54

**APPENDIX XI**: Signals used in aggressive interspecific interactions by family – page 55

**APPENDIX XII**: Supporting Information references – page 60

# APPENDIX I: Position categories for each component of posture in our dataset

| **TABLE S1:** Position categories for each component of posture, their definitions, and examples for nonpasserine and passerine birds in our dataset. | | | | | | | |
| --- | --- | --- | --- | --- | --- | --- | --- |
| **Categories** | **Definition** | **Nonpasserine examples** | | **Passerine examples** | | | |
|  |  | **Source** | **Species** | **Source** | | **Species** | |
| **body.orientation** |  |  |  |  | |  | |
| *forward.upright* | breast is raised, lower back lowered, so that the bird is more upright than typical when relaxed | <http://en.wikiaves.com/640519> | *Heliomaster squamosus* | <http://en.wikiaves.com/1876013> | | *Thraupis palmarum* | |
| *forward.lowered* | breast is lowered, lower back raised, such that the bird is flattened or even tipping downward | <http://en.wikiaves.com/766892&p=10&t=b> | *Galbula ruficauda* | <http://en.wikiaves.com/149494> | | *Dacnis cayana* | |
| *forward.normal* | body posture/orientation is typical of a relaxed individual (i.e., breast not raised or lowered) | <http://en.wikiaves.com/175322> | *Guira guira* | <http://en.wikiaves.com/1053815> | | *Thraupis cyanoptera* | |
| *side.oriented* | body is positioned to the side, such that the side is facing the receiver | <http://en.wikiaves.com/3288137> | *Columbina squammata* | <http://en.wikiaves.com/3232065> | | *Hylexetastes brigidai* | |
| *feet.forward* | body is positioned such that the feet are thrown forward, with the rest of the body positioned to facilitate this | <https://www.wikiaves.com.br/3137043> | *Rhynchotus rufescens* | <http://en.wikiaves.com/1195707> | | *Pitangus sulphuratus* | |
| *above* | body is positioned above the receiver, typically in the air | <http://en.wikiaves.com/1793062> | *Elanus leucurus* | <http://en.wikiaves.com/1188174> | | *Tyrannus savana* | |
| *upside.down* | body is positioned upside down during signal | <http://en.wikiaves.com/685015> | *Tapera naevia* |  | |  | |
|  |  |  |  |  | |  | |
| **head.position** |  |  |  |  | |  | |
| *forward.upright* | head is elevated relative to body, facing the receiver | <http://en.wikiaves.com/220548> | *Ardea cocoi* | <http://en.wikiaves.com/343686> | | *Thraupis palmarum* | |
| *foward.lowered* | head is lowered relative to body, facing the receiver | <http://en.wikiaves.com/2399766> | *Vanellus chilensis* | <http://en.wikiaves.com/736478> | | *Euphonia chalybea* | |
| *forward.normal* | head is in a position typical of a relaxed, perched individual, facing the receiver | <http://en.wikiaves.com/2399766> | *Phimosus infuscatus* | <http://en.wikiaves.com/668723> | | *Thraupis bonariensis* | |
| *side.oriented* | the side of the head is facing the receiver | <http://en.wikiaves.com/3288137> | *Columbina talpacoti* |  | |  | |
| *held.back.upward* | head is retreated backwards and upwards (toward the back) | <https://www.wikiaves.com.br/3137043> | *Rhynchotus rufescens* |  | |  | |
|  |  |  |  |  | |  | |
| **wing.position** |  |  |  |  | |  | |
| *flapping* | bird is actively flapping wings (including hummingbird hovering) | <http://en.wikiaves.com/325619> | *Clytolaema rubricauda* | <http://en.wikiaves.com/1166375> | | *Myiodynastes maculatus* | |
| *soaring.gliding* | bird is soaring or gliding | <http://en.wikiaves.com/885568> | *Falco peregrinus* |  | |  | |
| *spread.outward* | wings are spread outward from the body (horizontally) | <http://en.wikiaves.com/766892&p=10&t=b> | *Galbula ruficauda* | <http://en.wikiaves.com/685015> | | *Pitangus sulphuratus* | |
| *raised.upward* | wings are raised upward (vertically) above the level of the back | <http://en.wikiaves.com/184409> | *Chrysolampis mosquitus* | <http://en.wikiaves.com/2096823> | | *Euphonia pectoralis* | |
| *partially.spread* | wings are partially spread | <http://en.wikiaves.com/734377> | *Limosa haemastica* | <http://en.wikiaves.com/2097893> | | *Pitangus sulphuratus* | |
| *closed.flat* | wing are closed, sitting flat against the body | <http://en.wikiaves.com/2064415> | *Melanerpes flavifrons* | <http://en.wikiaves.com/2209202> | | *Pitangus sulphuratus* | |
| *closed.held.slightly.out* | wings are closed, held slightly out from the body | <http://en.wikiaves.com/708020> | *Thalasseus acuflavidus* | <http://en.wikiaves.com/1080456> | | *Thraupis sayaca* | |
| *closed.raised.off.back* | wings are closed, raised off the back (particularly the flight feathers) | <http://en.wikiaves.com/371061> | *Amazonetta brasiliensis* | <http://en.wikiaves.com/3215679> | | *Pitangus sulphuratus* | |
|  |  |  |  |  | |  | |
| **shoulder.position** |  |  |  |  | |  | |
| *underwing.forward* | wing is open/partially open, underwing is facing the receiver | <http://en.wikiaves.com/2658994> | *Pyrrhura frontalis* | <http://en.wikiaves.com/200437> | | *Cacicus chrysopterus* | |
| *upperwing.forward* | wing is open/partially open, upperwing is facing the receiver | <http://en.wikiaves.com/2352090> | *Plegadis chihi* | <http://en.wikiaves.com/1652714> | | *Spinus magellanica* | |
| *wing.horizontal (shoulder.forward.flight.*  *feathers.trailing)* | wing is open/partially open, held horizontally (shoulder is facing the receiver, flight feathers are trailing) | <http://en.wikiaves.com/1480656> | *Eupetomena macroura* | <http://en.wikiaves.com/552910> | | *Zonotrichia capensis* | |
| *wing.closed.shoulder.visible* | wing is closed, shoulder is exposed/visible | <http://en.wikiaves.com/726489> | *Eupsittula aurea* | <http://en.wikiaves.com/1196550> | | *Ramphocelus bresilia* | |
| *wing.closed.shoulder.concealed* | wing is closed, shoulder is concealed by breast feathers | <http://en.wikiaves.com/901664> | *Colaptes melanochloros* | <http://en.wikiaves.com/1000957> | | *Euphonia violacea* | |
|  |  |  |  |  | |  | |
| **bill.position** |  |  |  |  | |  | |
| *open.forward* | bill is open, facing the receiver | <http://en.wikiaves.com/1146211> | *Leucochloris albicollis* | <http://en.wikiaves.com/1967003> | | *Zimmerius acer* | |
| *open.upward* | bill is open, pointed upwards | [none] |  |  | |  | |
| *open.downward* | bill is open, pointed downwards | <http://en.wikiaves.com/763555> | *Phimosus infuscatus* |  | |  | |
| *open.side* | bill is open, held to the side relative to the receiver | <http://en.wikiaves.com/726489> | *Brotogeris chiriri* | <http://en.wikiaves.com/1532841> | | *Thraupis palmarum* | |
| *closed.forward* | bill is closed, facing the receiver | <http://en.wikiaves.com/1211846> | *Columbina minuta* | <http://en.wikiaves.com/2171672> | | *Cacicus chrysopterus* | |
| *closed.upward* | bill is closed, pointed upwards | <http://en.wikiaves.com/1043676> | *Tigrisoma lineatum* | <http://en.wikiaves.com/523772> | | *Pitangus sulphuratus* | |
| *closed.downward* | bill is closed, pointed downwards | <http://en.wikiaves.com/3101629> | *Egretta thula* |  | |  | |
| *closed.side* | bill is closed, held to the side relative to the receiver | <http://en.wikiaves.com/3288137> | *Columbina talpacoti* |  | |  | |
|  |  |  |  |  | |  | |
| **tail.position** |  |  |  |  | |  | |
| *trailing.fanned* | tail extends behind the body, is fanned | <http://en.wikiaves.com/1712472> | *Chloroceryle inda* | <http://en.wikiaves.com/1534449> | | *Tyrannus savana* | |
| *trailing.notfanned* | tail extends behind the body, is not fanned | <http://en.wikiaves.com/267309> | *Nystalus chacuru* | <http://en.wikiaves.com/1082497> | | *Pitangus sulphuratus* | |
| *raised.fanned* | tail is raised well above the level of the back (almost, or to the point of being, perpendicular), is fanned | <http://en.wikiaves.com/224111> | *Phalacrocorax brasilianus* | <http://en.wikiaves.com/393383> | | *Cacicus haemorhous* | |
| *raised.notfanned* | tail is raised well above the level of the back (almost, or to the point of being, perpendicular), is not fanned | <http://en.wikiaves.com/1116511> | *Calidris fuscicollis* |  | |  | |
| *down.fanned* | tail is held down, towards the belly, fanned | <http://en.wikiaves.com/701582> | *Eupetomena macroura* | <http://en.wikiaves.com/2508490> | | *Myiodynastes maculatus* | |
| *down.notfanned* | tail is held down, towards the belly, not fanned | <http://en.wikiaves.com/384131> | *Lophornis chalybeus* |  | |  | |
| *partlyraised.fanned* | tail is raised slightly above the level of the back, is fanned | <http://en.wikiaves.com/1239558> | *Theristicus caudatus* | <http://en.wikiaves.com/3232065> | | *Xiphorhynchus guttatus* | |
| *partlyraised.notfanned* | tail is raised slightly above the level of the back, is not fanned | <http://en.wikiaves.com/1211846> | *Columbina minuta* | <http://en.wikiaves.com/614107> | | *Thraupis sayaca* | |
| *side.fanned* | tail is held to the side, so that the side of the tail is facing the receiver, is fanned | <http://en.wikiaves.com/3288137> | *Columbina squammata* |  | |  | |
| *side.notfanned* | tail is held to the side, so that the side of the tail is facing the receiver, is not fanned | <http://en.wikiaves.com/231174> | *Tachyphonus coronatus* |  |  | |  |
|  |  |  |  |  | |  | |
| **feet.position** |  |  |  |  | |  | |
| *on.substrate* | feet are on a substrate (either on the ground, on a perch, or in the water) | <http://en.wikiaves.com/756200> | *Ara chloropterus* | <http://en.wikiaves.com/2168794> | | *Tangara peruviana* | |
| *tucked.up* | feet are tucked up against the belly | <http://en.wikiaves.com/701582> | *Eupetomena macroura* | <http://en.wikiaves.com/1549106> | | *Myiodynastes maculatus* | |
| *extended* | feet are extended outwards, towards the receiver | <http://en.wikiaves.com/158521> | *Geranoaetus albicaudatus* | <http://en.wikiaves.com/3266139> | | *Molothrus bonariensis* | |
| *partly.extended* | feet are partially extended outwards, towards the receiver | <http://en.wikiaves.com/1933441> | *Anodorhynchus hyacinthinus* | <http://en.wikiaves.com/968506> | | *Pitangus sulphuratus* | |
| *hanging* | feet are dangling below the belly, are not being extended out towards the receiver | <http://en.wikiaves.com/734377> | *Pluvialis squatorola* | <http://en.wikiaves.com/2097893> | | *Turdus rufiventris* | |
|  |  |  |  |  | |  | |
| **closest.point** |  |  |  |  | |  | |
| *bill* | the closest point to the receiver is the bill | <http://en.wikiaves.com/855123> | *Leucochloris albicollis* | <http://en.wikiaves.com/2141052> | | *Thraupis cyanoptera* | |
| *feet* | the closest point to the receiver is the feet | <http://en.wikiaves.com/1769786> | *Caracara plancus* |  | |  | |
| *wing* | the closest point to the receiver is the wings | <http://en.wikiaves.com/3288137> | *Columbina talpacoti* |  | |  | |
| *tail* | the closest point to the receiver is the tail | [none] |  |  | |  | |
| *breast* | the closest point to the receiver is the breast | <http://en.wikiaves.com/1886997> | *Penelope jacquacu* |  | |  | |

# APPENDIX II: Instructions for naïve observers examining images for body regions and colors most highlighted in interspecific aggressive signals

Please review the photos and videos (links provided) that illustrate two species of birds

interacting. Please identify the body regions that the focal individual highlights most in their

signal towards the other species. You can select up to 3 body regions. Body regions are defined

in the file called "bird_topography". Please also identify the most highlighted colors in the

signal towards the other species. You can select up to 3. See below for a list of color

categories. Please enter your data in the excel file provided, by typing the number 1 in the

appropriate columns.

**Most highlighted region(s) of the body used in the signal** (pick no more than 3)**:**

mouth

bill

face/throat

breast

belly

sides

crown

back/nape

shoulders

upperwings

underwings

uppertail.coverts

uppertail

undertail

undertail.coverts

tarsal.feathers

legs/feet

**Most highlighted colors used in the** **signal** (pick no more than 3)**:**

red/pink/orange/yellow

black

white

dark/white contrast

blue/green/violet

rufous/chestnut

brown/beige

gray

# APPENDIX III: Color names considered to be synonymous with each focal color or color group

| **Table S2:** Color names considered to be part of each focal color or color group | |
| --- | --- |
| **Focal color/color group** | **Color names included** |
| carotenoids (red, pink, orange, yellow) | red, pink, orange, yellow |
| black | black |
| white | white, ivory |
| rufous/chestnut | rufous, chestnut, cinnamon |
| brown/beige | brown, beige, tan, buff, flesh |
| gray | gray, dusky |
| black/dark and white contrast | white *and* black, fuscous, dark, dusky, or dark gray |
| blue/green/violet | blue, green, violet |

# APPENDIX IV: Phylogeny of species included in dataset


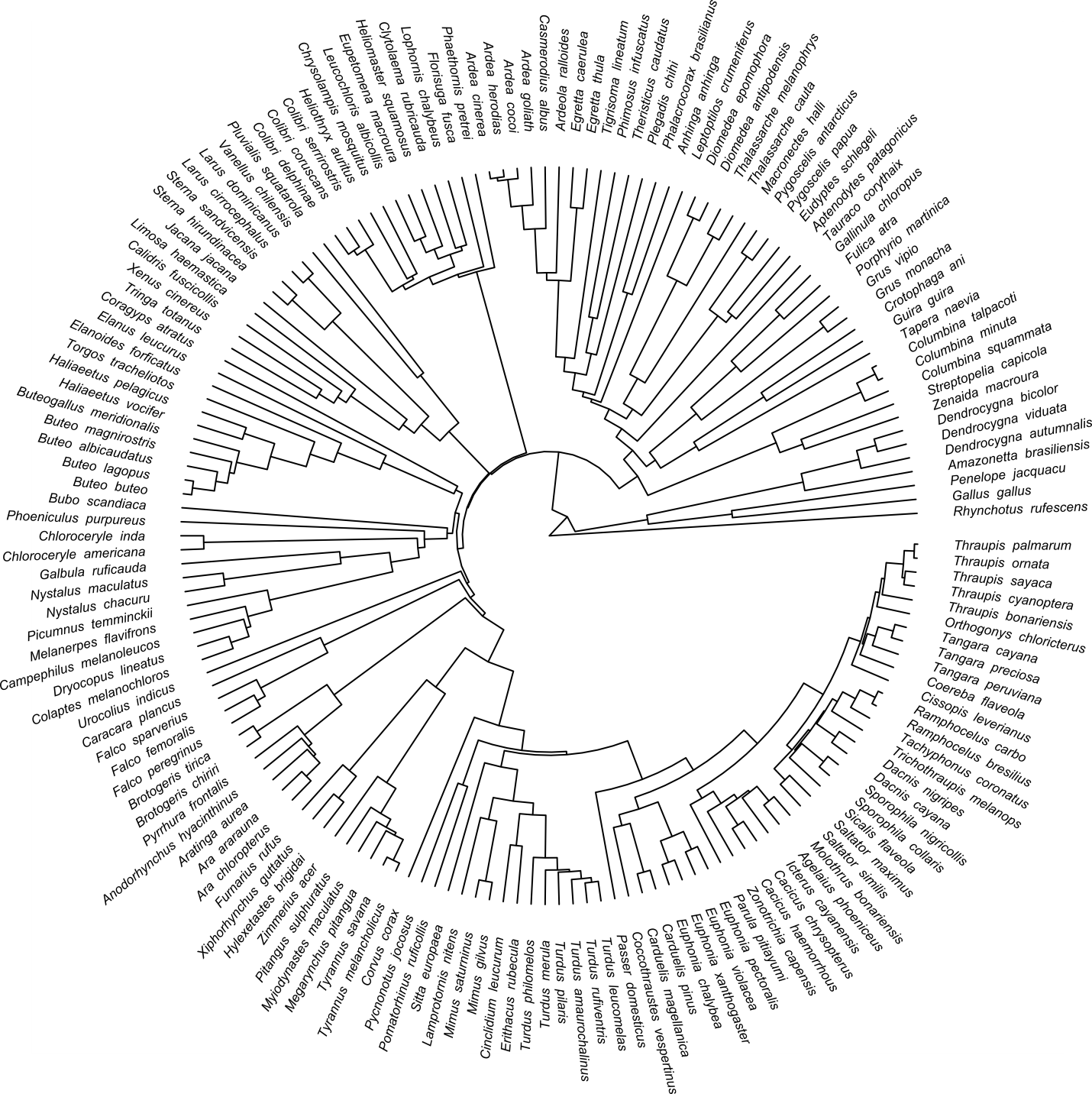


**Figure S1:** Phylogeny of the 164 species included in our dataset from Jetz et al. (2012).

# APPENDIX V: Posture model performance, pairwise comparisons, and diagnostics

| **Table S3.** Performance of models explaining variation in postures assumed in interspecific aggressive interactions, after controlling for the effects of phylogeny using a Bayesian generalized linear mixed model approach (MCMCglmm). Models with lower DIC scores are considered to be better performing. | |
| --- | --- |
|  | |
| **model** | **DIC** |
| **Body orientation** | |
| posture.assumed ~ body.orientation | 1638.49 |
| posture.assumed ~ 1 (null) | 1973.08 |
|  | |
| **Head position** | |
| posture.assumed ~ head.position | 1355.38 |
| posture.assumed ~ 1 (null) | 1721.35 |
|  | |
| **Wing position** | |
| posture.assumed ~ wing.position | 1968.29 |
| posture.assumed ~ 1 (null) | 2067.74 |
|  | |
| **Wing position: closed vs. open** | |
| posture.assumed ~ wings.open | 970.89 |
| posture.assumed ~ 1 (null) | 977.77 |
|  |  |
| **Shoulder position** | |
| posture.assumed ~ shoulder.position | 1484.29 |
| posture.assumed ~ 1 (null) | 1721.41 |
|  | |
| **Bill position** | |
| posture.assumed ~ bill.position | 1072.10 |
| posture.assumed ~ 1 (null) | 1886.01 |
|  | |
| **Tail position** | |
| posture.assumed ~ tail.position | 1536.15 |
| posture.assumed ~ 1 (null) | 2022.94 |
|  | |
| **Feet position** | |
| posture.assumed ~ feet.position | 890.03 |
| posture.assumed ~ 1 (null) | 1690.17 |
|  | |
| **Closest point** | |
| posture.assumed ~ closest.point | 881.73 |
| posture.assumed ~ 1 (null) | 2454.68 |
|  | |

| **Table S4.** Pairwise comparisons between body orientations assumed in aggressive encounters between heterospecifics (*N* = 337 interspecific interactions). Values are *pMCMC* results from the best performing model explaining variation in body orientations assumed in interspecific aggressive interactions, after controlling for the effects of phylogeny using a Bayesian generalized linear mixed model approach (MCMCglmm). *pMCMC* values < 0.05 indicate significant differences in how commonly two body orientations are used. See Figure 3a for effect sizes. | | | | | | | |
| --- | --- | --- | --- | --- | --- | --- | --- |
|  |  |  |  |  |  |  |  |
|  | forward.lowered | above | feet.forward | forward.normal | forward.upright | side.oriented | upside.down |
| forward.lowered |  |  |  |  |  |  |  |
| above | <0.001 |  |  |  |  |  |  |
| feet.forward | <0.001 | 0.29 |  |  |  |  |  |
| forward.normal | <0.001 | <0.001 | <0.001 |  |  |  |  |
| forward.upright | <0.001 | <0.001 | <0.001 | <0.001 |  |  |  |
| side.oriented | <0.001 | 0.47 | 0.08 | <0.001 | <0.001 |  |  |
| upside.down | <0.001 | 0.54 | 0.65 | <0.001 | <0.001 | 0.18 |  |
|  |  |  |  |  |  |  |  |

| **Table S5.** Pairwise comparisons between head positions assumed in aggressive encounters between heterospecifics (*N* = 336 interspecific interactions). Values are *pMCMC* results from the best performing model explaining variation in head positions assumed in interspecific aggressive interactions, after controlling for the effects of phylogeny using a Bayesian generalized linear mixed model approach (MCMCglmm). *pMCMC* values < 0.05 indicate significant differences in how commonly two head positions are used. See Figure 3b for effect sizes. | | | | | |
| --- | --- | --- | --- | --- | --- |
|  |  |  |  |  |  |
|  | forward.lowered | forward.upright | forward.normal | side.oriented | held.back.upward |
| forward.lowered |  |  |  |  |  |
| forward.upright | <0.001 |  |  |  |  |
| forward.normal | <0.001 | 0.50 |  |  |  |
| side.oriented | <0.001 | <0.001 | <0.001 |  |  |
| held.back.upward | <0.001 | <0.001 | <0.001 | 0.001 |  |
|  |  |  |  |  |  |

| **Table S6.** Pairwise comparisons between wing positions assumed in aggressive encounters between heterospecifics (*N* = 337 interspecific interactions). Values are *pMCMC* results from the best performing models explaining variation in wing positions assumed in interspecific aggressive interactions, after controlling for the effects of phylogeny using a Bayesian generalized linear mixed model approach (MCMCglmm). *pMCMC* values < 0.05 indicate significant differences in how commonly two wing positions are used. See Figure 3c for effect sizes. | | | | | | | | |
| --- | --- | --- | --- | --- | --- | --- | --- | --- |
|  |  |  |  |  |  |  |  |  |
|  | closed.held.slightly.out | closed.flat | closed.raised.off.back | flapping | spread.outward | partially.spread | raised.upward | soaring.gliding |
| closed.held.slightly.out |  |  |  |  |  |  |  |  |
| closed.flat | 0.10 |  |  |  |  |  |  |  |
| closed.raised.off.back | <0.001 | <0.001 |  |  |  |  |  |  |
| flapping | 0.12 | 0.91 | <0.001 |  |  |  |  |  |
| spread.outward | 0.12 | 0.91 | <0.001 | 1.00 |  |  |  |  |
| partially.spread | <0.001 | <0.001 | 0.06 | <0.001 | <0.001 |  |  |  |
| raised.upward | <0.001 | <0.001 | 0.06 | <0.001 | <0.001 | 1.00 |  |  |
| soaring.gliding | <0.001 | <0.001 | 0.23 | <0.001 | <0.001 | 0.002 | 0.003 |  |
|  |  |  |  |  |  |  |  |  |
| **b) Wing position: closed vs. open** |  |  |  |  |  |  |  |  |
|  | closed | | | open | | | | |
| closed |  | | |  | | | | |
| open | 0.004 | | |  | | | | |
|  |  |  |  |  |  |  |  |  |

| **Table S7.** Pairwise comparisons between shoulder positions assumed in aggressive encounters between heterospecifics (*N* = 336 interspecific interactions). Values are *pMCMC* results from the best performing model explaining variation in shoulder positions assumed in interspecific aggressive interactions, after controlling for the effects of phylogeny using a Bayesian generalized linear mixed model approach (MCMCglmm). *pMCMC* values < 0.05 indicate significant differences in how commonly two shoulder positions are used. See Figure 3d for effect sizes. | | | | | |
| --- | --- | --- | --- | --- | --- |
|  |  |  |  |  |  |
|  | underwing.forward | wing.closed.  shoulder.visible | wing.horizontal | wing.closed.  shoulder.concealed | upperwing.forward |
| underwing forward |  |  |  |  |  |
| wing.closed.shoulder.visible | 0.42 |  |  |  |  |
| wing.horizontal | <0.001 | <0.001 |  |  |  |
| wing.closed.shoulder.concealed | <0.001 | <0.001 | 0.49 |  |  |
| upperwing.forward | <0.001 | <0.001 | 0.11 | 0.35 |  |
|  |  |  |  |  |  |

| **Table S8.** Pairwise comparisons between bill positions assumed in aggressive encounters between heterospecifics (*N* = 316 interspecific interactions). Values are *pMCMC* results from the best performing model explaining variation in bill positions assumed in interspecific aggressive interactions, after controlling for the effects of phylogeny using a Bayesian generalized linear mixed model approach (MCMCglmm). *pMCMC* values < 0.05 indicate significant differences in how commonly two bill positions are used. See Figure 3e for effect sizes. | | | | | | | |
| --- | --- | --- | --- | --- | --- | --- | --- |
|  |  |  |  |  |  |  |  |
|  | open.forward | closed.forward | open.side | closed.side | closed.downward | open.downward | closed.upward |
| open.forward |  |  |  |  |  |  |  |
| closed.forward | <0.001 |  |  |  |  |  |  |
| open.side | <0.001 | <0.001 |  |  |  |  |  |
| closed.side | <0.001 | <0.001 | 0.99 |  |  |  |  |
| closed.downward | <0.001 | <0.001 | 0.23 | 0.21 |  |  |  |
| open.downward | <0.001 | <0.001 | 0.12 | 0.11 | 0.67 |  |  |
| closed.upward | <0.001 | <0.001 | 0.04 | 0.04 | 0.39 | 0.62 |  |
|  |  |  |  |  |  |  |  |

| **Table S9.** Pairwise comparisons between tail positions assumed in aggressive encounters between heterospecifics (*N* = 295 interspecific interactions). Values are *pMCMC* results from the best performing model explaining variation in tail positions assumed in interspecific aggressive interactions, after controlling for the effects of phylogeny using a Bayesian generalized linear mixed model approach (MCMCglmm). *pMCMC* values < 0.05 indicate significant differences in how commonly two tail positions are used. See Figure 3f for effect sizes. | | | | | | | | | | |
| --- | --- | --- | --- | --- | --- | --- | --- | --- | --- | --- |
|  |  |  |  |  |  |  |  |  |  |  |
|  | trailing.notfanned | trailing.fanned | down.fanned | down.notfanned | raised.fanned | partlyraised.  notfanned | partlyraised.  fanned | raised.notfanned | side.fanned | side.notfanned |
| trailing.notfanned |  |  |  |  |  |  |  |  |  |  |
| trailing.fanned | <0.001 |  |  |  |  |  |  |  |  |  |
| down.fanned | <0.001 | <0.001 |  |  |  |  |  |  |  |  |
| down.notfanned | <0.001 | <0.001 | <0.001 |  |  |  |  |  |  |  |
| raised.fanned | <0.001 | <0.001 | <0.001 | 0.83 |  |  |  |  |  |  |
| partlyraised.notfanned | <0.001 | <0.001 | <0.001 | 0.50 | 0.65 |  |  |  |  |  |
| partlyraised.fanned | <0.001 | <0.001 | <0.001 | 0.23 | 0.33 | 0.59 |  |  |  |  |
| raised.notfanned | <0.001 | <0.001 | <0.001 | 0.07 | 0.11 | 0.26 | 0.55 |  |  |  |
| side.fanned | <0.001 | <0.001 | <0.001 | 0.03 | 0.05 | 0.14 | 0.35 | 0.73 |  |  |
| side.notfanned | <0.001 | <0.001 | <0.001 | <0.001 | <0.001 | 0.003 | 0.02 | 0.07 | 0.13 |  |
|  |  |  |  |  |  |  |  |  |  |  |

| **Table S10.** Pairwise comparisons between feet positions assumed in aggressive encounters between heterospecifics (*N* = 336 interspecific interactions). Values are *pMCMC* results from the best performing model explaining variation in feet positions assumed in interspecific aggressive interactions, after controlling for the effects of phylogeny using a Bayesian generalized linear mixed model approach (MCMCglmm). *pMCMC* values < 0.05 indicate significant differences in how commonly two feet positions are used. See Figure 3g for effect sizes. | | | | | |
| --- | --- | --- | --- | --- | --- |
|  |  |  |  |  |  |
|  | on.substrate | extended | tucked.up | partly.extended | hanging |
| on.substrate |  |  |  |  |  |
| extended | <0.001 |  |  |  |  |
| tucked.up | <0.001 | <0.001 |  |  |  |
| partly.extended | <0.001 | <0.001 | 0.82 |  |  |
| hanging | <0.001 | <0.001 | 0.12 | 0.21 |  |
|  |  |  |  |  |  |

| **Table S11.** Pairwise comparisons between the body region positioned closest to the competitor in aggressive encounters between heterospecifics (*N* = 337 interspecific interactions). Values are *pMCMC* results from the best performing model explaining variation in the body region positioned closest to the competitor in interspecific aggressive interactions, after controlling for the effects of phylogeny using a Bayesian generalized linear mixed model approach (MCMCglmm). *pMCMC* values < 0.05 indicate significant differences in how commonly two body regions are held closest to the competitor. See Figure 3h for effect sizes. | | | | | | | | | | | | | | |
| --- | --- | --- | --- | --- | --- | --- | --- | --- | --- | --- | --- | --- | --- | --- |
|  |  |  |  |  |  |  |  |  |  |  |  |  |  |  |
|  | bill | bill.wing | bill.feet | bill.neck | bill.forehead | bill.face | bill.head | bill.throat.neck | bill.tail | feet | wing | breast | feet.tail | side |
| bill |  |  |  |  |  |  |  |  |  |  |  |  |  |  |
| bill.wing | <0.001 |  |  |  |  |  |  |  |  |  |  |  |  |  |
| bill.feet | <0.001 | 0.72 |  |  |  |  |  |  |  |  |  |  |  |  |
| bill.neck | <0.001 | 0.22 | 0.36 |  |  |  |  |  |  |  |  |  |  |  |
| bill.forehead | <0.001 | 0.06 | 0.13 | 0.48 |  |  |  |  |  |  |  |  |  |  |
| bill.face | <0.001 | 0.07 | 0.13 | 0.49 | 0.96 |  |  |  |  |  |  |  |  |  |
| bill.head | <0.001 | 0.06 | 0.12 | 0.49 | 0.99 | 0.98 |  |  |  |  |  |  |  |  |
| bill.throat.neck | <0.001 | 0.07 | 0.12 | 0.49 | 0.96 | 0.99 | 0.99 |  |  |  |  |  |  |  |
| bill.tail | <0.001 | 0.22 | 0.37 | 0.99 | 0.50 | 0.51 | 0.50 | 0.49 |  |  |  |  |  |  |
| feet | <0.001 | <0.001 | <0.001 | <0.001 | <0.001 | <0.001 | <0.001 | <0.001 | <0.001 |  |  |  |  |  |
| wing | <0.001 | 0.38 | 0.22 | 0.04 | 0.008 | 0.009 | 0.008 | 0.008 | 0.03 | <0.001 |  |  |  |  |
| breast | <0.001 | 0.22 | 0.37 | 0.99 | 0.50 | 0.52 | 0.48 | 0.48 | 0.99 | <0.001 | 0.04 |  |  |  |
| feet.tail | <0.001 | 0.06 | 0.13 | 0.48 | 0.99 | 0.99 | 0.99 | 0.97 | 0.51 | <0.001 | 0.01 | 0.51 |  |  |
| side | <0.001 | 0.07 | 0.12 | 0.51 | 0.97 | 0.98 | 0.99 | 0.99 | 0.49 | <0.001 | 0.006 | 0.49 | 0.98 |  |
|  |  |  |  |  |  |  |  |  |  |  |  |  |  |  |

**
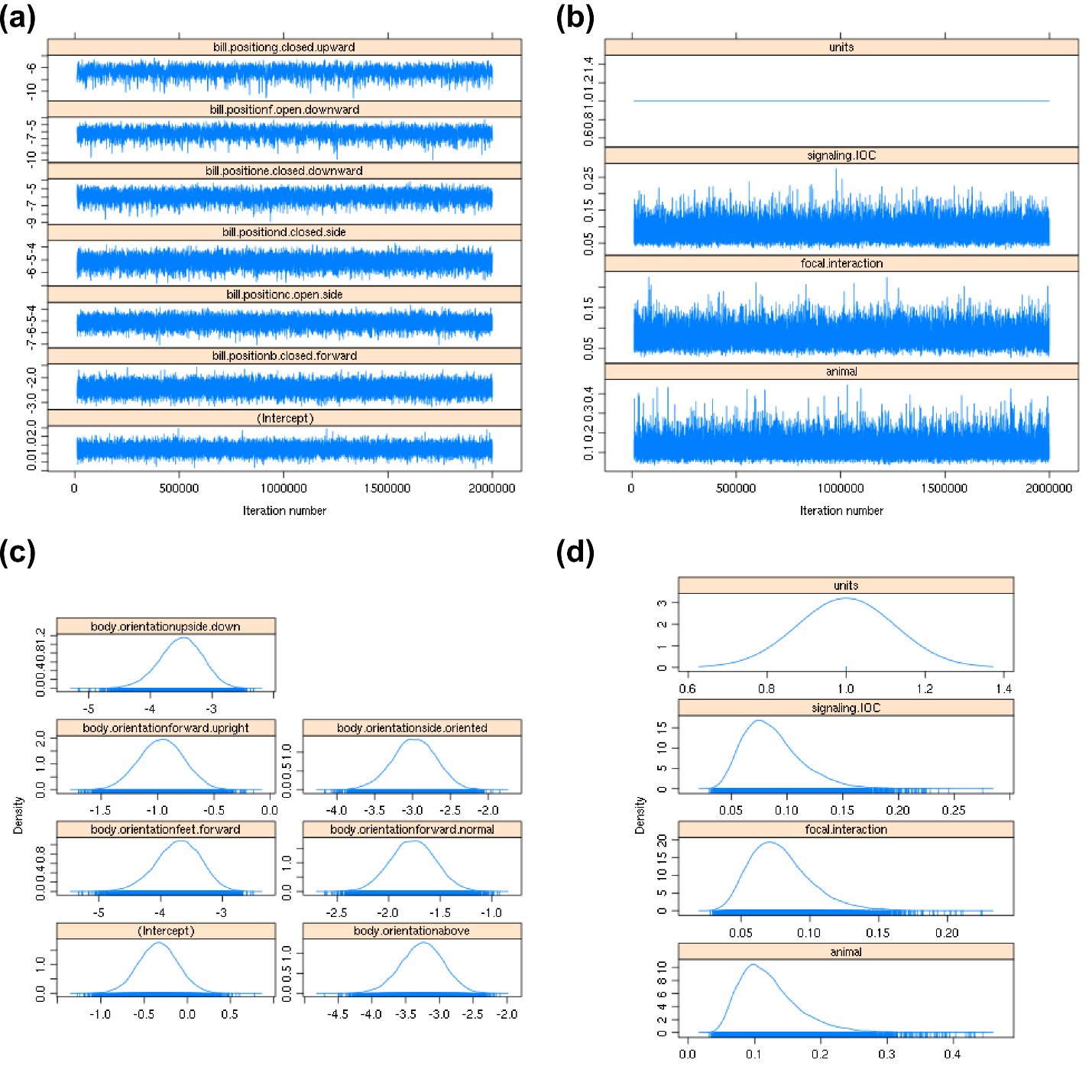
**

**Figure S2: Diagnostic plots for body orientation MCMCglmm analysis indicate model convergence:** a) trace plots for fixed effects show no pattern across iterations; b) trace plots for random effects show no patterns across iterations; c) density plots for fixed effects show smooth, approximately normal distributions; d) density plots for random effects show smooth distributions.

**
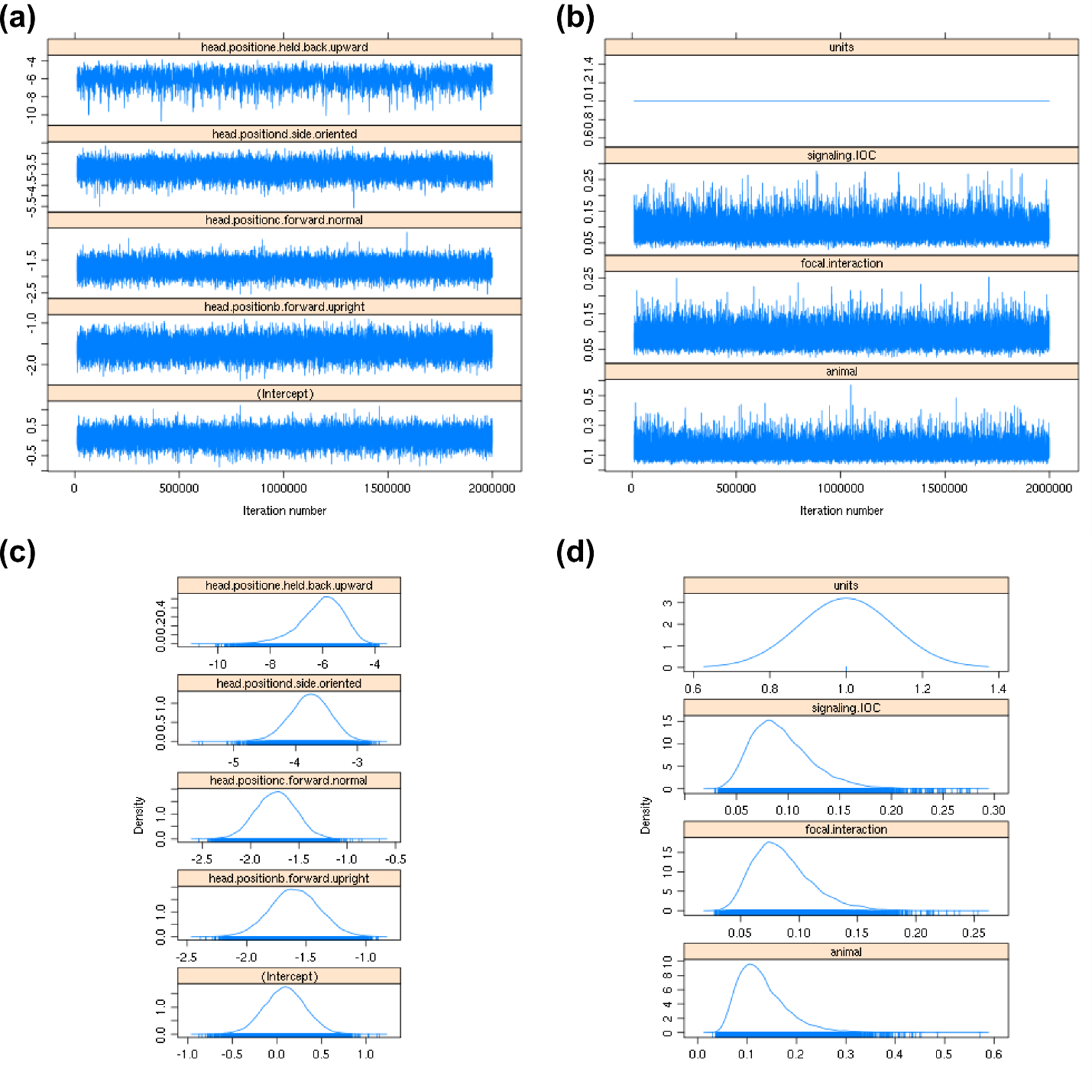
**

**Figure S3: Diagnostic plots for head position MCMCglmm analysis indicate model convergence:** a) trace plots for fixed effects show no pattern across iterations; b) trace plots for random effects show no patterns across iterations; c) density plots for fixed effects show smooth, approximately normal distributions; d) density plots for random effects show smooth distributions.


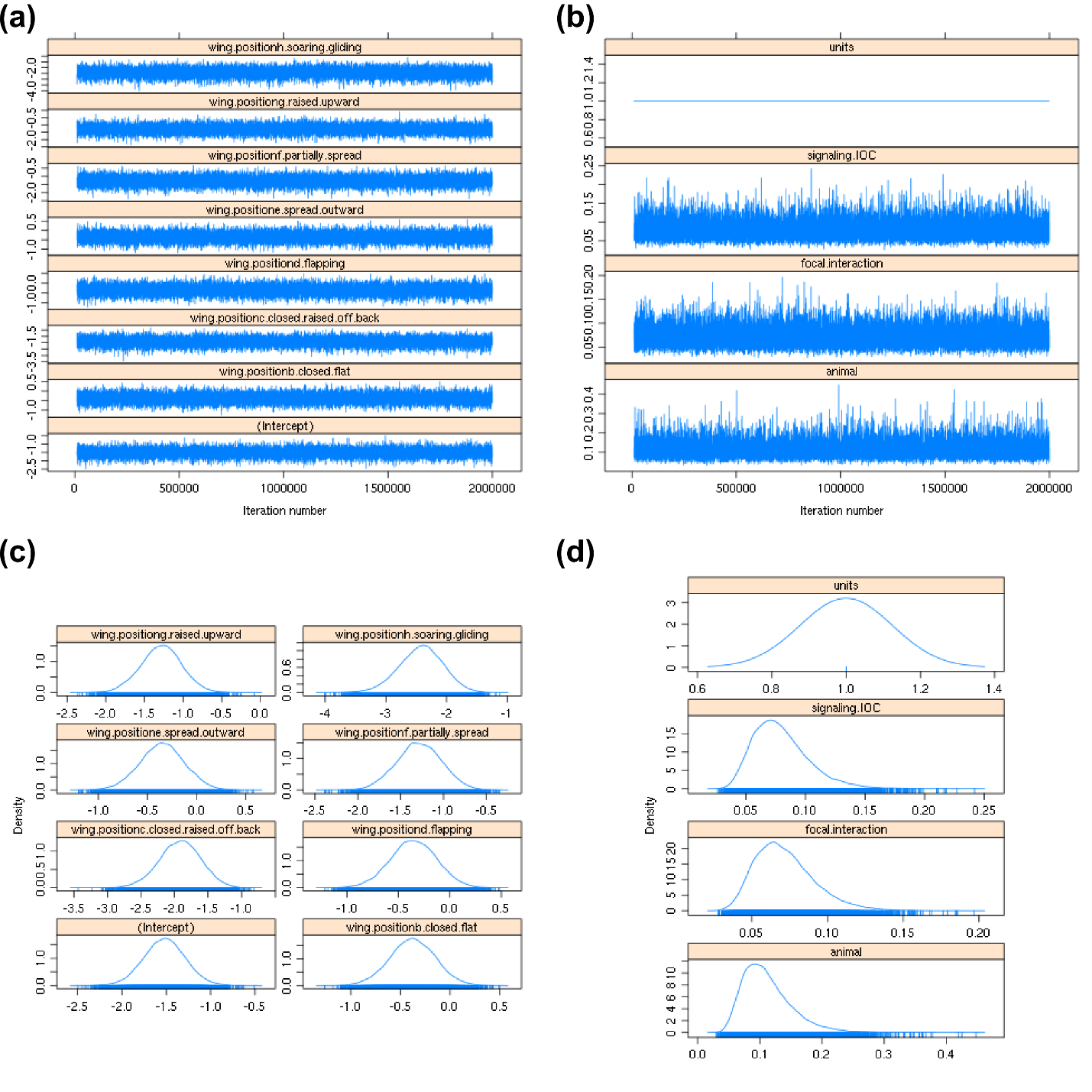


**Figure S4: Diagnostic plots for wing position MCMCglmm analysis indicate model convergence:** a) trace plots for fixed effects show no pattern across iterations; b) trace plots for random effects show no patterns across iterations; c) density plots for fixed effects show smooth, approximately normal distributions; d) density plots for random effects show smooth distributions.


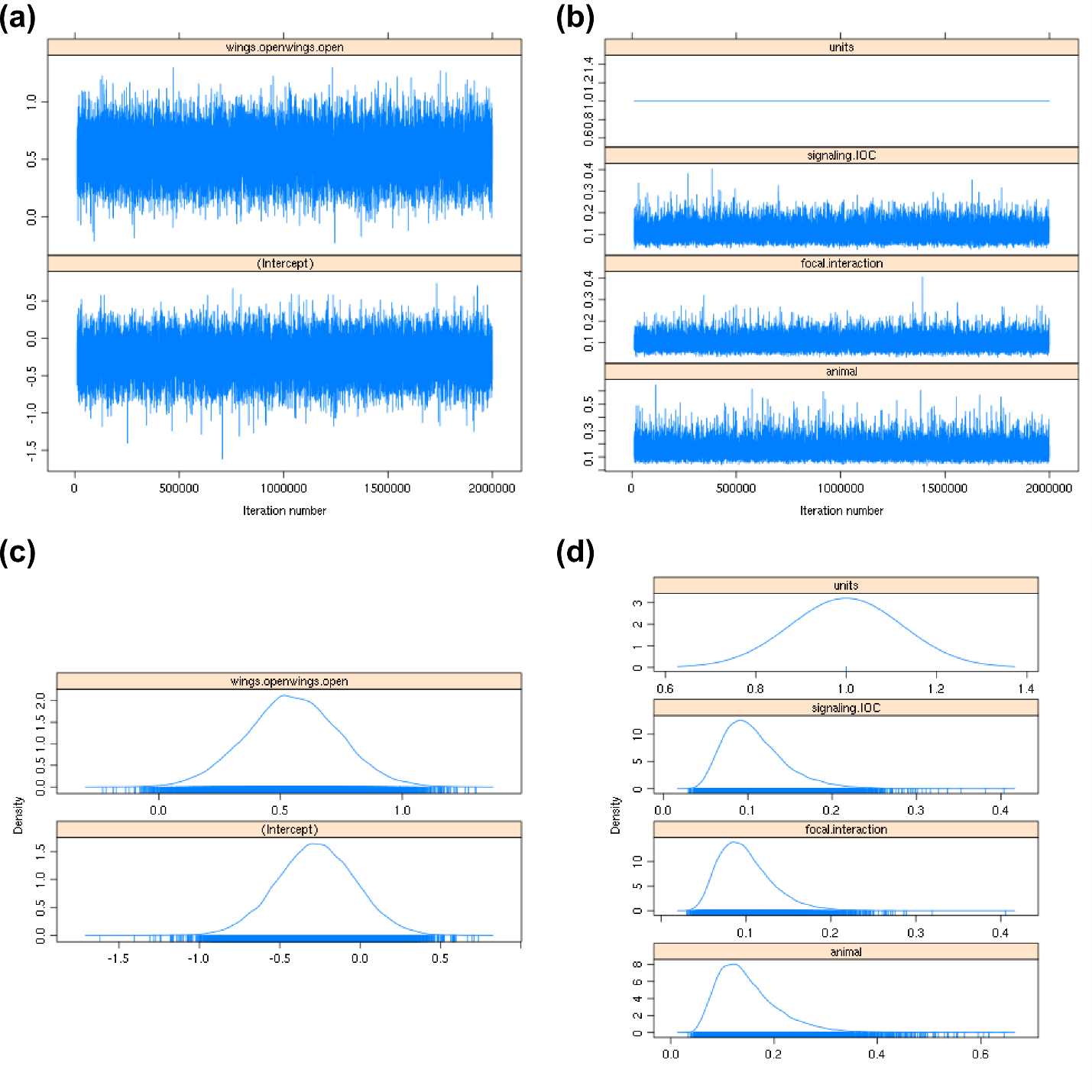


**Figure S5: Diagnostic plots for wing position: open vs. closed MCMCglmm analysis indicate model convergence:** a) trace plots for fixed effects show no pattern across iterations; b) trace plots for random effects show no patterns across iterations; c) density plots for fixed effects show smooth, approximately normal distributions; d) density plots for random effects show smooth distributions.


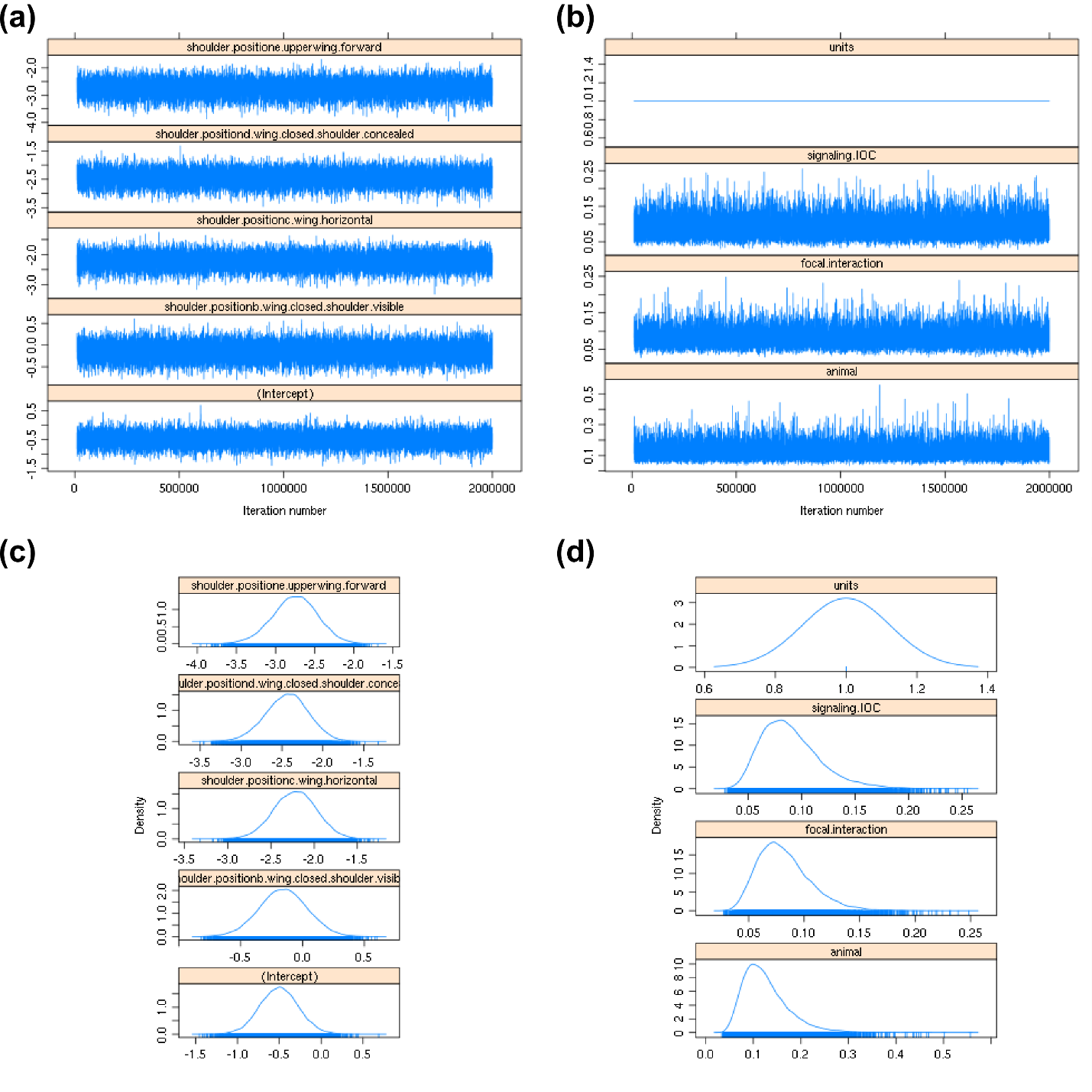


**Figure S6: Diagnostic plots for shoulder position MCMCglmm analysis indicate model convergence:** a) trace plots for fixed effects show no pattern across iterations; b) trace plots for random effects show no patterns across iterations; c) density plots for fixed effects show smooth, approximately normal distributions; d) density plots for random effects show smooth distributions.


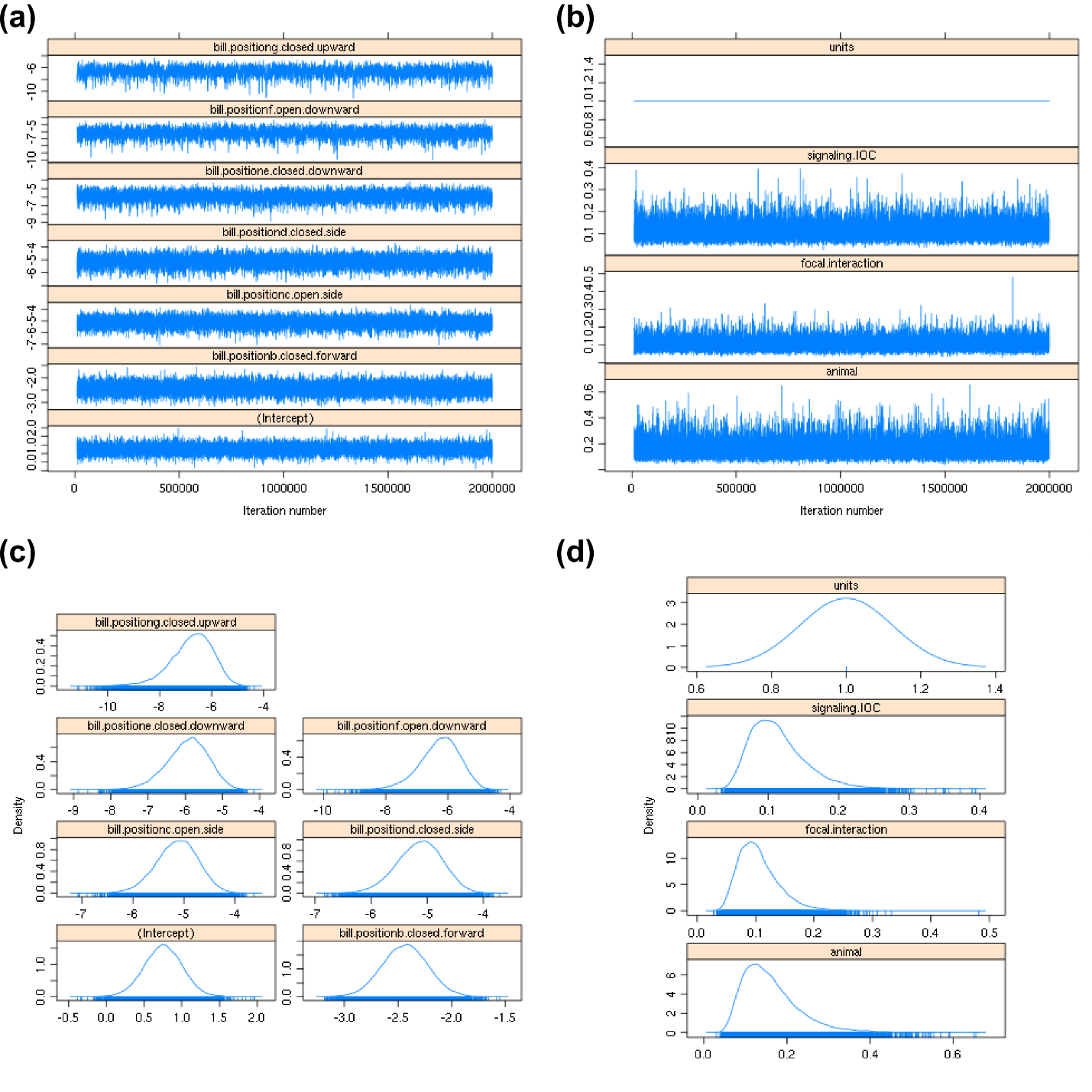


**Figure S7: Diagnostic plots for bill position MCMCglmm analysis indicate model convergence:** a) trace plots for fixed effects show no pattern across iterations; b) trace plots for random effects show no patterns across iterations; c) density plots for fixed effects show smooth, approximately normal distributions; d) density plots for random effects show smooth distributions.


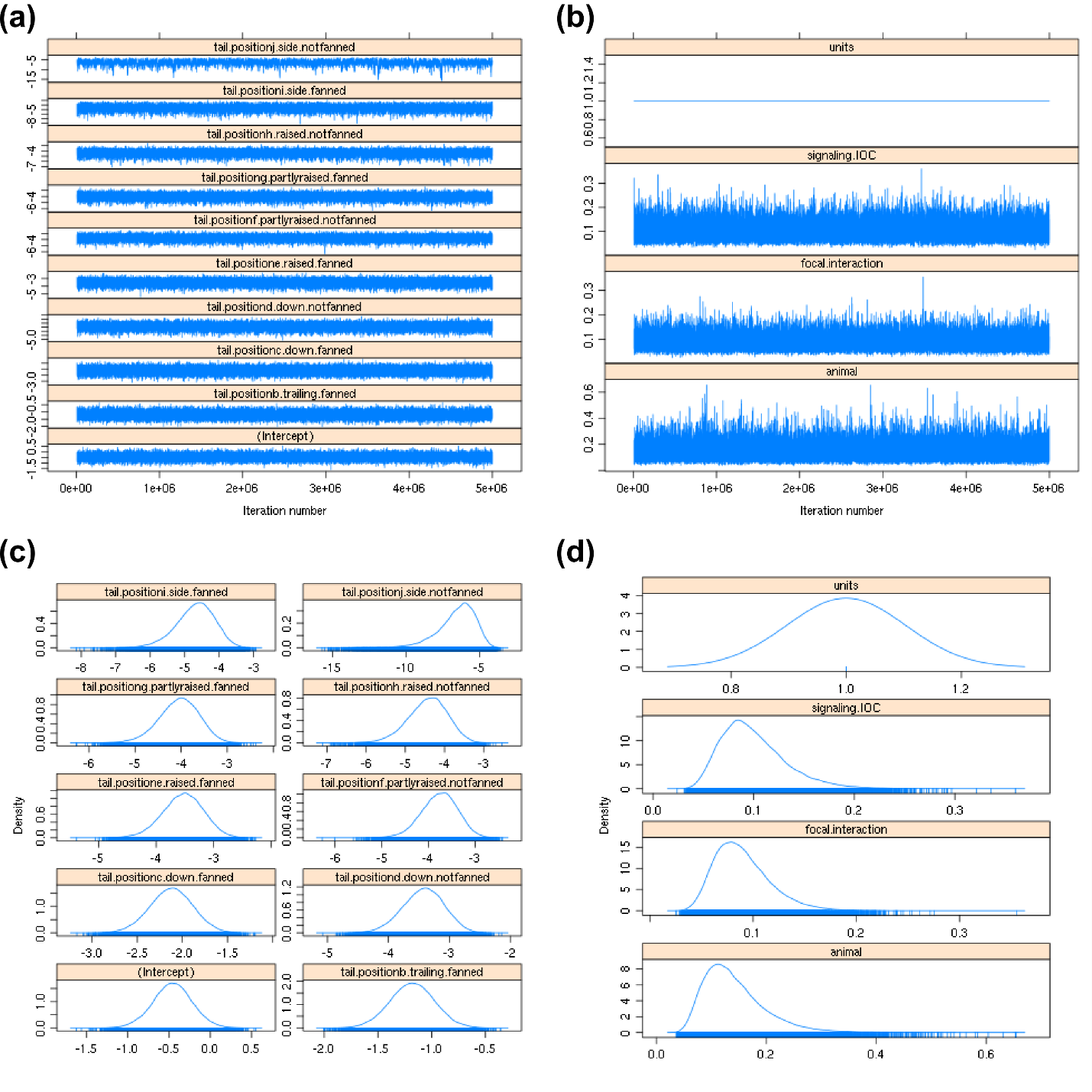


**Figure S8: Diagnostic plots for tail position MCMCglmm analysis indicate model convergence:** a) trace plots for fixed effects show no pattern across iterations; b) trace plots for random effects show no patterns across iterations; c) density plots for fixed effects show smooth, approximately normal distributions; d) density plots for random effects show smooth distributions.


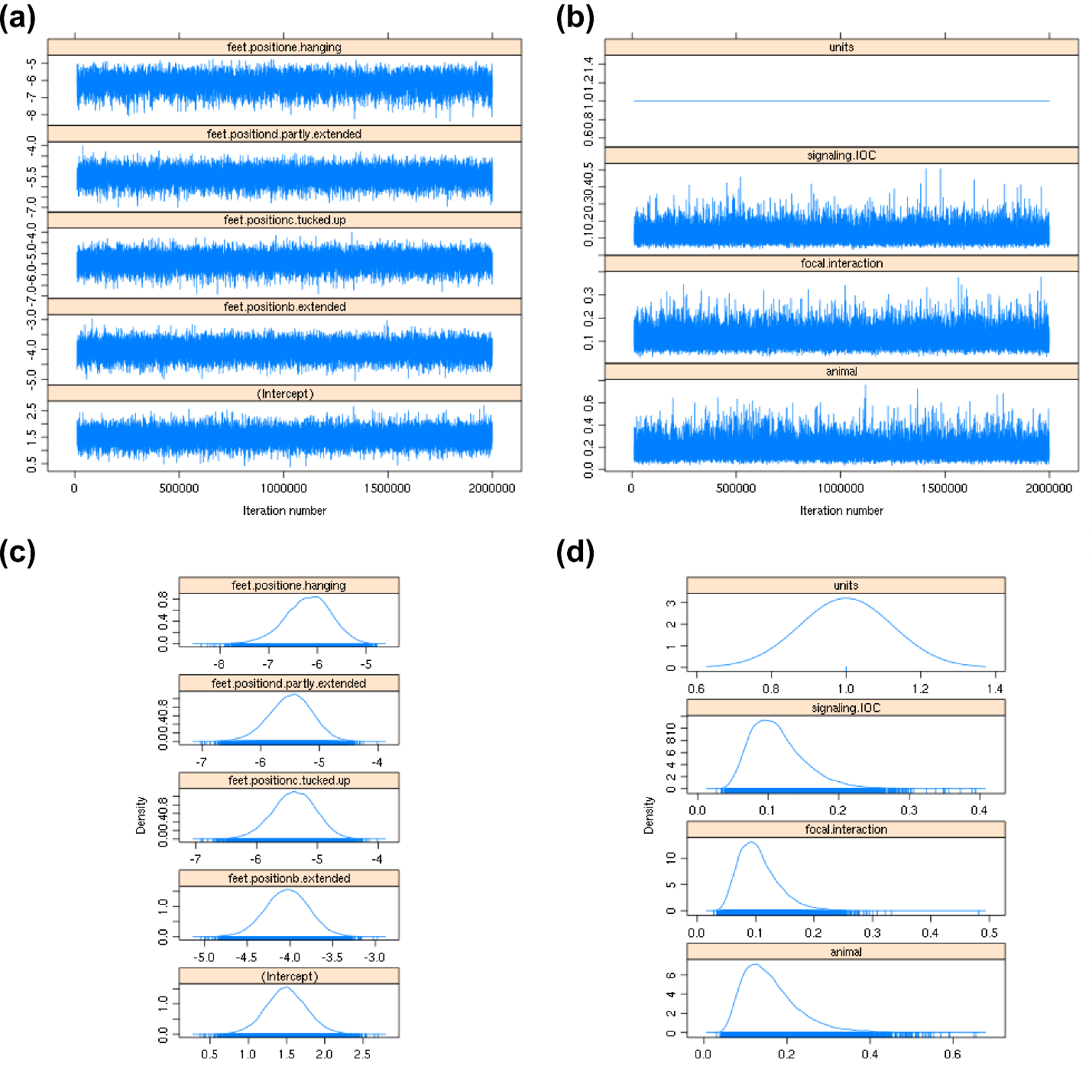


**Figure S9: Diagnostic plots for feet position MCMCglmm analysis indicate model convergence:** a) trace plots for fixed effects show no pattern across iterations; b) trace plots for random effects show no patterns across iterations; c) density plots for fixed effects show smooth, approximately normal distributions; d) density plots for random effects show smooth distributions.


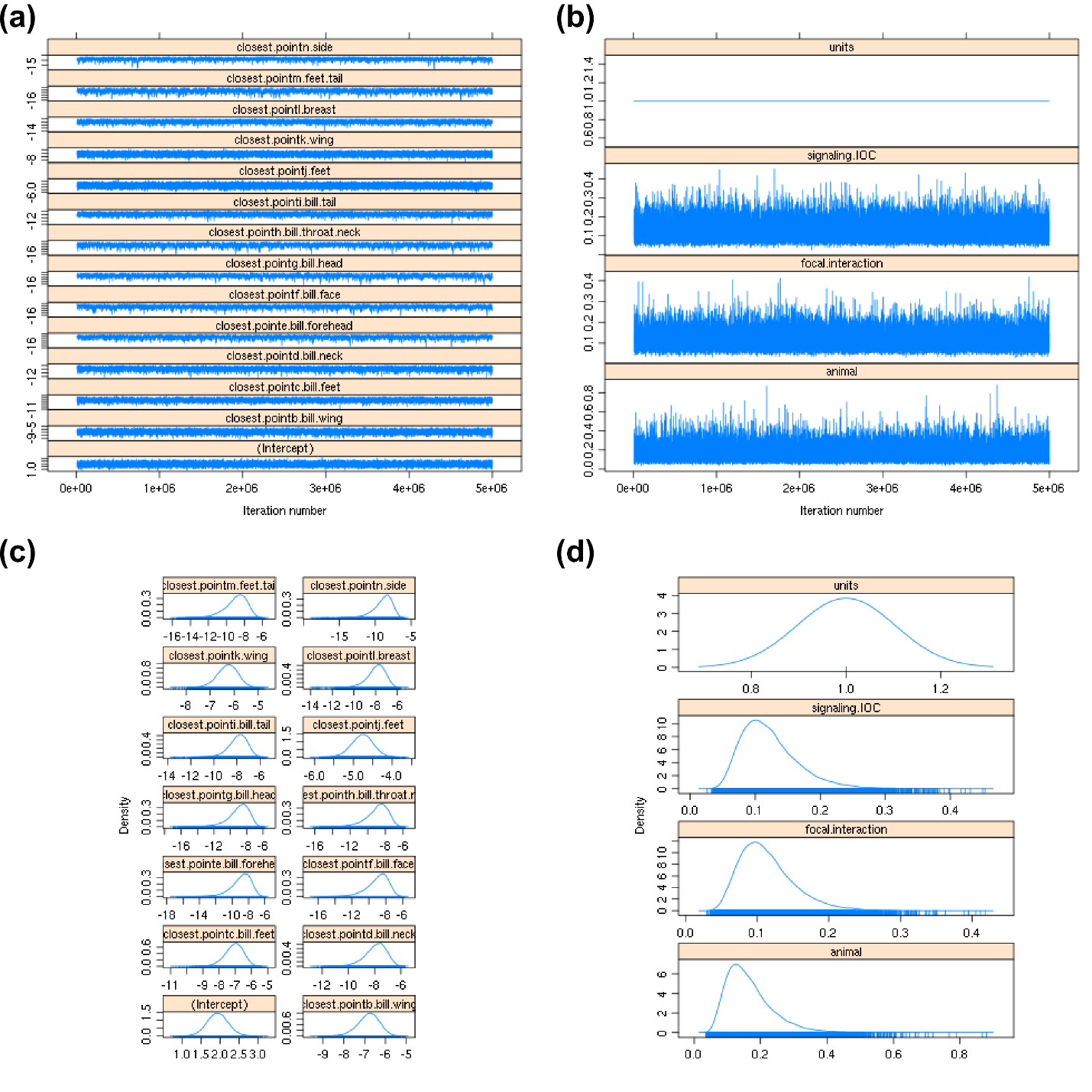


**Figure S10: Diagnostic plots for closest point MCMCglmm analysis indicate model convergence:** a) trace plots for fixed effects show no pattern across iterations; b) trace plots for random effects show no patterns across iterations; c) density plots for fixed effects show smooth, approximately normal distributions; d) density plots for random effects show smooth distributions.

| **Table S12:** Diagnostic values for assessing model fit for MCMCglmm analyses examining postures used in aggressive interspecific interactions. | | | |
| --- | --- | --- | --- |
|  | | | |
| 1. **Body orientation** | | | |
|  | *Upper confidence interval for Gelman and Rubin’s Convergence Diagnostic*^a^ | *Effective sample size*^b^ | *Geweke’s Convergence Diagnostic*^c^ |
| Intercept: forward.lowered | 1 | 19900 | 0.53 |
| above | 1 | 12733 | 0.31 |
| feet.forward | 1 | 8997 | 1.05 |
| forward.normal | 1 | 19141 | -0.98 |
| forward.upright | 1 | 19900 | 0.76 |
| side.oriented | 1 | 14078 | 0.19 |
| upside.down | 1 | 9703 | -0.52 |
|  |  |  |  |
| 1. **Head position** | | | |
|  | *Upper confidence interval for Gelman and Rubin’s Convergence Diagnostic*^a^ | *Effective sample size*^b^ | *Geweke’s Convergence Diagnostic*^c^ |
| Intercept: forward.lowered | 1 | 19900 | -1.82 |
| forward.upright | 1 | 17959 | 0.38 |
| forward.normal | 1 | 19900 | -0.14 |
| side.oriented | 1 | 11674 | 0.71 |
| held.back.upward | 1 | 1514 | 0.21 |
|  |  |  |  |
| 1. **Wing position** | | | |
|  | *Upper confidence interval for Gelman and Rubin’s Convergence Diagnostic*^a^ | *Effective sample size*^b^ | *Geweke’s Convergence Diagnostic*^c^ |
| Intercept: closed.held.slightly.out | 1 | 19900 | -2.06 |
| closed.flat | 1 | 19900 | 2.39 |
| closed.raised.off.back | 1 | 14146 | 0.74 |
| flapping | 1 | 19900 | 1.26 |
| spread.outward | 1 | 19900 | 0.34 |
| partially.spread | 1 | 17760 | 0.14 |
| raised.upward | 1 | 17756 | -0.003 |
| soaring.gliding | 1 | 10389 | 0.001 |
|  |  |  |  |
| 1. **Wing position: closed vs. open** | | | |
|  | *Upper confidence interval for Gelman and Rubin’s Convergence Diagnostic*^a^ | *Effective sample size*^b^ | *Geweke’s Convergence Diagnostic*^c^ |
| Intercept: closed | 1 | 19900 | 0.06 |
| open | 1 | 20431 | -0.04 |
|  |  |  |  |
| 1. **Shoulder position** | | | |
|  | *Upper confidence interval for Gelman and Rubin’s Convergence Diagnostic*^a^ | *Effective sample size*^b^ | *Geweke’s Convergence Diagnostic*^c^ |
| Intercept: underwing forward | 1 | 19900 | -0.22 |
| wing.closed.shoulder.visible | 1 | 19900 | -0.28 |
| wing.horizontal | 1 | 17524 | 0.10 |
| wing.closed.shoulder.concealed | 1 | 16140 | 0.88 |
| upperwing.forward | 1 | 15342 | 0.13 |
|  |  |  |  |
| 1. **Bill position** | | | |
|  | *Upper confidence interval for Gelman and Rubin’s Convergence Diagnostic*^a^ | *Effective sample size*^b^ | *Geweke’s Convergence Diagnostic*^c^ |
| Intercept: open.forward | 1 | 19900 | -1.03 |
| closed.forward | 1 | 19308 | 1.05 |
| open.side | 1 | 7704 | 1.02 |
| closed.side | 1 | 7537 | 0.35 |
| closed.downward | 1 | 3831 | -0.19 |
| open.downward | 1 | 2647 | 0.39 |
| closed.upward | 1 | 1523 | 1.54 |
|  |  |  |  |
| 1. **Tail position** | | | |
|  | *Upper confidence interval for Gelman and Rubin’s Convergence Diagnostic*^a^ | *Effective sample size*^b^ | *Geweke’s Convergence Diagnostic*^c^ |
| Intercept: trailing.notfanned | 1 | 49900 | -1.92 |
| trailing.fanned | 1 | 49131 | 1.78 |
| down.fanned | 1 | 45342 | 0.24 |
| down.notfanned | 1 | 27738 | 1.69 |
| raised.fanned | 1 | 16769 | -0.34 |
| partlyraised.notfanned | 1 | 20952 | -0.99 |
| partlyraised.fanned | 1 | 16769 | 2.44 |
| raised.notfanned | 1 | 11395 | 0.99 |
| side.fanned | 1 | 8718 | 0.74 |
| side.notfanned | 1 | 1249 | 0.58 |
|  |  |  |  |
| 1. **Feet position** | | | |
|  | *Upper confidence interval for Gelman and Rubin’s Convergence Diagnostic*^a^ | *Effective sample size*^b^ | *Geweke’s Convergence Diagnostic*^c^ |
| Intercept: on.substrate | 1 | 19663 | 0.56 |
| extended | 1 | 18513 | -0.20 |
| tucked.up | 1 | 10171 | -1.02 |
| partly.extended | 1 | 10172 | 0.24 |
| hanging | 1 | 5444 | 1.40 |
|  |  |  |  |
| 1. **Closest point** | | | |
|  | *Upper confidence interval for Gelman and Rubin’s Convergence Diagnostic*^a^ | *Effective sample size*^b^ | *Geweke’s Convergence Diagnostic*^c^ |
| Intercept: bill | 1.00 | 46585 | -0.98 |
| bill.wing | 1.00 | 11553 | 0.56 |
| bill.feet | 1.00 | 44041 | 0.10 |
| bill.neck | 1.00 | 3723 | 0.80 |
| bill.forehead | 1.00 | 1488 | 2.58 |
| bill.face | 1.00 | 1390 | -0.50 |
| bill.head | 1.01 | 1268 | 2.20 |
| bill.throat.neck | 1.01 | 1413 | -0.36 |
| feet | 1.00 | 44041 | 1.28 |
| wing | 1.00 | 11553 | 0.17 |
| breast | 1.00 | 3856 | 1.08 |
| feet.tail | 1.00 | 1565 | 0.01 |
| side | 1.00 | 1395 | 0.11 |
|  |  |  |  |
| ^a^ values approaching 1 indicate convergence across three runs of our model (Bolker et al. 2012); calculated using the *gelman.diag* function in the *coda* R package (Plummer et al. 2016) | | | |
| ^b^ effective sample sizes exceeding 200 are acceptable for reliable confidence intervals (Bolker et al. 2012) | | | |
| ^c^ values between -1.96 and 1.96 indicate convergence; calculated using the *geweke.diag* function in the *coda* R package (Plummer et al. 2016) | | | |

# APPENDIX VI: Posture model performance and pairwise comparisons from subset of data obtained from photographs

| **Table S13.** Performance of models explaining variation in postures assumed in interspecific aggressive interactions in subset of data obtained from photographs, after controlling for the effects of phylogeny using a Bayesian generalized linear mixed model approach (MCMCglmm). Models with lower DIC scores are considered to be better performing. | |
| --- | --- |
|  | |
| **model** | **DIC** |
| **Body orientation** | |
| posture.assumed ~ body.orientation | 1406.96 |
| posture.assumed ~ 1 (null) | 1695.51 |
|  | |
| **Head position** | |
| posture.assumed ~ head.position | 1156.27 |
| posture.assumed ~ 1 (null) | 1478.39 |
|  | |
| **Wing position** | |
| posture.assumed ~ wing.position | 1686.83 |
| posture.assumed ~ 1 (null) | 1776.40 |
|  | |
| **Wing position: closed vs. open** | |
| posture.assumed ~ wings.open | 838.35 |
| posture.assumed ~ 1 (null) | 841.28 |
|  |  |
| **Shoulder position** | |
| posture.assumed ~ shoulder.position | 1267.47 |
| posture.assumed ~ 1 (null) | 1478.35 |
|  | |
| **Bill position** | |
| posture.assumed ~ bill.position | 899.02 |
| posture.assumed ~ 1 (null) | 1611.55 |
|  | |
| **Tail position** | |
| posture.assumed ~ tail.position | 1315.79 |
| posture.assumed ~ 1 (null) | 1727.66 |
|  | |
| **Feet position** | |
| posture.assumed ~ feet.position | 805.97 |
| posture.assumed ~ 1 (null) | 1446.83 |
|  | |
| **Closest point** | |
| posture.assumed ~ closest.point | 770.86 |
| posture.assumed ~ 1 (null) | 2108.86 |
|  | |

# APPENDIX VII: Highlighted body regions and highlighted colors model performance, pairwise comparisons, and diagnostics

| **Table S14.** Performance of models explaining variation in body regions and colors highlighted in interspecific aggressive interactions, after controlling for the effects of phylogeny using a Bayesian generalized linear mixed model approach (MCMCglmm). Models with lower DIC scores are considered to be better performing. | |
| --- | --- |
|  | |
| **model** | **DIC** |
| **Highlighted body regions** | |
| highlighted ~ region | 3413.20 |
| highlighted ~ 1 (null) | 4458.48 |
|  | |
| **Highlighted colors** | |
| highlighted ~ color | 2536.37 |
| highlighted ~ 1 (null) | 2688.91 |
|  | |

| **Table S15.** Pairwise comparisons between body regions highlighted in aggressive encounters between heterospecifics (*N* = 337 interspecific interactions). Values are *pMCMC* results from the best performing model explaining variation in body regions highlighted in interspecific aggressive interactions, after controlling for the effects of phylogeny using a Bayesian generalized linear mixed model approach (MCMCglmm). *pMCMC* values < 0.05 indicate significant differences in how commonly two body regions are highlighted. See Figure 4a for effect sizes. | | | | | | | | | | | | | | |
| --- | --- | --- | --- | --- | --- | --- | --- | --- | --- | --- | --- | --- | --- | --- |
|  |  |  |  |  |  |  |  |  |  |  |  |  |  |  |
|  | face.throat | mouth | underwings | bill | breast | nape.back | legs.feet | crown | undertail | upperwings | belly | uppertail | shoulders | sides |
| face.throat |  |  |  |  |  |  |  |  |  |  |  |  |  |  |
| mouth | <0.001 |  |  |  |  |  |  |  |  |  |  |  |  |  |
| underwings | <0.001 | 0.02 |  |  |  |  |  |  |  |  |  |  |  |  |
| bill | <0.001 | 0.01 | 0.91 |  |  |  |  |  |  |  |  |  |  |  |
| breast | <0.001 | <0.001 | <0.001 | <0.001 |  |  |  |  |  |  |  |  |  |  |
| nape.back | <0.001 | <0.001 | <0.001 | <0.001 | 0.09 |  |  |  |  |  |  |  |  |  |
| legs.feet | <0.001 | <0.001 | <0.001 | <0.001 | 0.15 | 0.47 |  |  |  |  |  |  |  |  |
| crown | <0.001 | <0.001 | <0.001 | <0.001 | 0.008 | 0.32 | 0.77 |  |  |  |  |  |  |  |
| undertail | <0.001 | <0.001 | <0.001 | <0.001 | <0.001 | 0.009 | 0.06 | 0.11 |  |  |  |  |  |  |
| upperwings | <0.001 | <0.001 | <0.001 | <0.001 | <0.001 | 0.01 | 0.06 | 0.12 | 0.99 |  |  |  |  |  |
| belly | <0.001 | <0.001 | <0.001 | <0.001 | <0.001 | 0.002 | 0.01 | 0.04 | 0.61 | 0.63 |  |  |  |  |
| uppertail | <0.001 | <0.001 | <0.001 | <0.001 | <0.001 | <0.001 | 0.003 | 0.008 | 0.29 | 0.29 | 0.58 |  |  |  |
| shoulders | <0.001 | <0.001 | <0.001 | <0.001 | <0.001 | <0.001 | <0.001 | <0.001 | 0.06 | 0.07 | 0.17 | 0.42 |  |  |
| sides | <0.001 | <0.001 | <0.001 | <0.001 | <0.001 | <0.001 | <0.001 | <0.001 | <0.001 | <0.001 | <0.001 | 0.002 | 0.02 |  |
|  |  |  |  |  |  |  |  |  |  |  |  |  |  |  |

| **Table S16.** Pairwise comparisons between colors highlighted in aggressive encounters between heterospecifics (*N* = 337 interspecific interactions). Values are *pMCMC* results from the best performing model explaining variation in colors highlighted in interspecific aggressive interactions, after controlling for the effects of phylogeny using a Bayesian generalized linear mixed model approach (MCMCglmm). *pMCMC* values < 0.05 indicate significant differences in how commonly two colors are highlighted. See Figure 4b for effect sizes. | | | | | | | | |
| --- | --- | --- | --- | --- | --- | --- | --- | --- |
|  |  |  |  |  |  |  |  |  |
|  | red.pink.  orange.yellow | blue.green.violet | black | dark.white.contrast | brown.beige | gray | white | rufous.chestnut |
| red.pink.orange.yellow |  |  |  |  |  |  |  |  |
| blue.green.violet | 0.02 |  |  |  |  |  |  |  |
| black | <0.001 | 0.02 |  |  |  |  |  |  |
| dark.white.contrast | <0.001 | 0.02 | 0.98 |  |  |  |  |  |
| brown.beige | <0.001 | <0.001 | 0.13 | 0.16 |  |  |  |  |
| gray | <0.001 | <0.001 | <0.001 | <0.001 | 0.05 |  |  |  |
| white | <0.001 | <0.001 | <0.001 | <0.001 | <0.001 | 0.12 |  |  |
| rufous.chestnut | <0.001 | <0.001 | <0.001 | <0.001 | <0.001 | 0.03 | 0.29 |  |


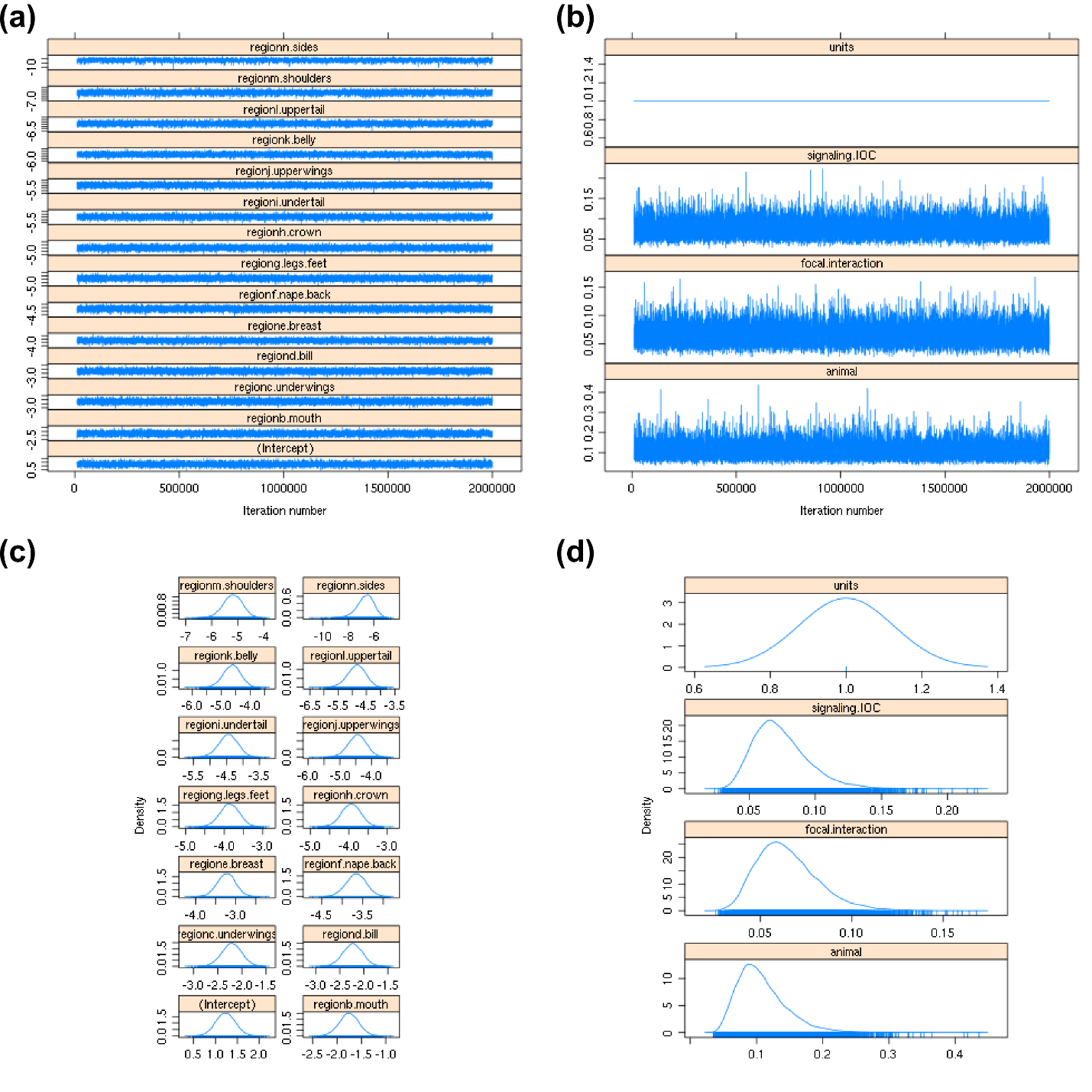


**Figure S11: Diagnostic plots for highlighted body regions MCMCglmm analysis indicate model convergence:** a) trace plots for fixed effects show no pattern across iterations; b) trace plots for random effects show no patterns across iterations; c) density plots for fixed effects show smooth, approximately normal distributions; d) density plots for random effects show smooth distributions.

| **Table S17:** Diagnostic values for assessing model fit for MCMCglmm analyses examining body regions highlighted in aggressive interspecific interactions | | | |
| --- | --- | --- | --- |
|  | *Upper confidence interval for Gelman and Rubin’s Convergence Diagnostic*^a^ | *Effective sample size*^b^ | *Geweke’s Convergence Diagnostic* ^c^ |
| Intercept: face.throat.bill | 1 | 19900 | -1.36 |
| mouth | 1 | 19900 | 0.93 |
| underwings | 1 | 19436 | 1.55 |
| bill | 1 | 19900 | 1.87 |
| breast | 1 | 18823 | 0.21 |
| nape.back | 1 | 18743 | 1.52 |
| legs.feet | 1 | 18151 | -1.13 |
| crown | 1 | 19727 | -0.57 |
| undertail | 1 | 15927 | 1.35 |
| upperwings | 1 | 15818 | 0.34 |
| belly | 1 | 14225 | 0.94 |
| uppertail | 1 | 12801 | -0.06 |
| shoulders | 1 | 10385 | 0.68 |
| sides | 1 | 2752 | 1.07 |
|  |  |  |  |
| ^a^ values approaching 1 indicate convergence across three runs of our model (Bolker et al. 2012); calculated using the *gelman.diag* function in the *coda* R package (Plummer et al. 2016) | | | |
| ^b^ effective sample sizes exceeding 200 are acceptable for reliable confidence intervals (Bolker et al. 2012) | | | |
| ^c^ values between -1.96 and 1.96 indicate convergence; calculated using the *geweke.diag* function in the *coda* R package (Plummer et al. 2016) | | | |

**
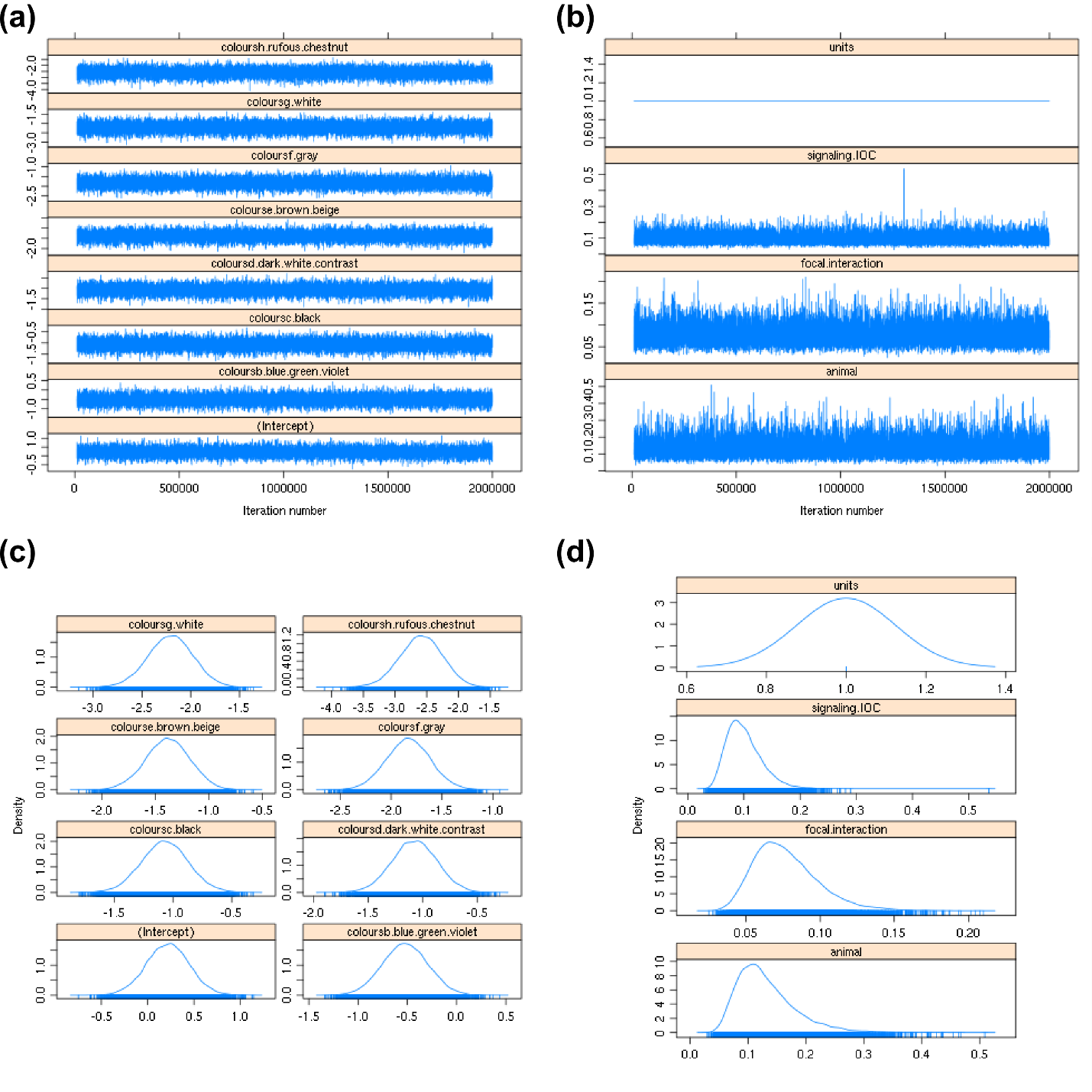
**

**Figure S12: Diagnostic plots for highlighted colors MCMCglmm analysis indicate model convergence:** a) trace plots for fixed effects show no pattern across iterations; b) trace plots for random effects show no patterns across iterations; c) density plots for fixed effects show smooth, approximately normal distributions; d) density plots for random effects show smooth distributions.

| **Table S18:** Diagnostic values for assessing model fit for MCMCglmm analyses examining colors highlighted in aggressive interspecific interactions | | | |
| --- | --- | --- | --- |
|  | *Upper confidence interval for Gelman and Rubin’s Convergence Diagnostic* ^a^ | *Effective sample size*^b^ | *Geweke’s Convergence Diagnostic* ^c^ |
| Intercept: red.pink.orange.yellow | 1 | 20211 | 1.21 |
| blue.green.violet | 1 | 19900 | -0.79 |
| black | 1 | 18986 | -0.83 |
| dark.white.contrast | 1 | 19402 | -1.20 |
| brown.beige | 1 | 18624 | -0.79 |
| gray | 1 | 19083 | -1.71 |
| white | 1 | 19247 | -1.01 |
| rufous.chestnut | 1 | 17887 | -1.27 |
|  |  |  |  |
| ^a^ values approaching 1 indicate convergence across three runs of our model (Bolker et al. 2012); calculated using the *gelman.diag* function in the *coda* R package (Plummer et al. 2016) | | | |
| ^b^ effective sample sizes exceeding 200 are acceptable for reliable confidence intervals (Bolker et al. 2012) | | | |
| ^c^ values between -1.96 and 1.96 indicate convergence; calculated using the *geweke.diag* function in the *coda* R package (Plummer et al. 2016) | | | |

# APPENDIX VIII: Highlighted body regions and colors model performance and pairwise comparisons from subset of data obtained from photographs

| **Table S19.** Performance of models explaining variation in body regions and colors highlighted in interspecific aggressive interactions in subset of data obtained from photographs, after controlling for the effects of phylogeny using a Bayesian generalized linear mixed model approach (MCMCglmm). Models with lower DIC scores are considered to be better performing. | |
| --- | --- |
|  | |
| **model** | **DIC** |
| **Highlighted body regions** | |
| highlighted ~ region | 2911.35 |
| highlighted ~ 1 (null) | 3842.62 |
|  | |
| **Highlighted colors** | |
| highlighted ~ color | 2155.54 |
| highlighted ~ 1 (null) | 2297.04 |
|  | |

# APPENDIX IX: Degree of similarity to intraspecific aggressive signal model performance, pairwise comparisons, and diagnostics

| **Table S20.** Performance of models explaining variation in the degree of similarity between in interspecific aggressive interactions and intraspecific aggressive interactions, after controlling for the effects of phylogeny using a Bayesian generalized linear mixed model approach (MCMCglmm). Models with lower DIC scores are considered to be better performing. | |
| --- | --- |
|  | |
| **model** | **DIC** |
| **Similarity between signals used in interspecific and intraspecific aggressive interactions** | |
| assumed ~ similarity | 681.96 |
| assumed ~ 1 (null) | 1424.31 |
|  | |

| **Table S21.** Pairwise comparisons between degrees of similarity between visual signals used interspecific aggressive interactions and intraspecific aggressive interactions (*N* = 307 interspecific interactions). Values are *pMCMC* results from the best performing model explaining variation in the degree of similarity between visual signals used interspecific aggressive interactions and intraspecific aggressive interactions, after controlling for the effects of phylogeny using a Bayesian generalized linear mixed model approach (MCMCglmm). *pMCMC* values < 0.05 indicate significant differences in how commonly two degrees of similarity are observed. See main text of Results for further details. | | | | |
| --- | --- | --- | --- | --- |
|  |  |  |  |  |
|  | same | similar | similar to congener | different |
| same |  |  |  |  |
| similar | <0.001 |  |  |  |
| similar to congener | <0.001 | <0.001 |  |  |
| different | <0.001 | <0.001 | 0.99 |  |
|  |  |  |  |  |


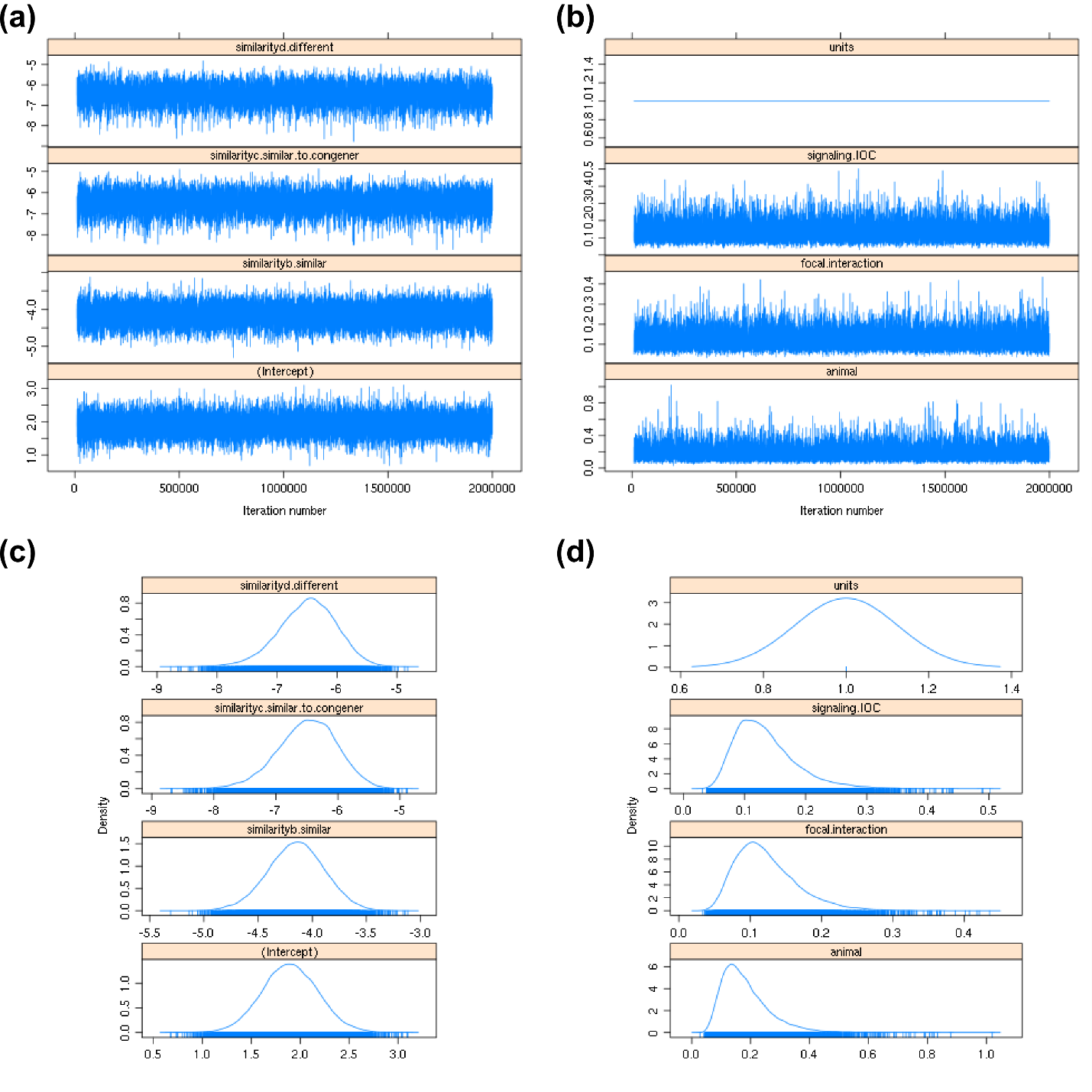


**Figure S13: Diagnostic plots for degree of similarity to intraspecific signals MCMCglmm analysis indicate model convergence:** a) trace plots for fixed effects show no pattern across iterations; b) trace plots for random effects show no patterns across iterations; c) density plots for fixed effects show smooth, approximately normal distributions; d) density plots for random effects show smooth distributions.

| **Table S22:** Diagnostic values for assessing model fit for main MCMCglmm analysis examining degree of similarity to intraspecific aggressive signal | | | |
| --- | --- | --- | --- |
|  | *Upper confidence interval for Gelman and Rubin’s Convergence Diagnostic*^a^ | *Effective sample size*^b^ | *Geweke’s Convergence Diagnostic* ^c^ |
| Intercept: same | 1 | 19432 | -0.97 |
| similar | 1 | 18959 | -0.77 |
| similar to congener | 1 | 6268 | 0.38 |
| different | 1 | 6434 | 1.41 |
|  |  |  |  |
| ^a^ values approaching 1 indicate convergence across three runs of our model (Bolker et al. 2012); calculated using the *gelman.diag* function in the *coda* R package (Plummer et al. 2016) | | | |
| ^b^ effective sample sizes exceeding 200 are acceptable for reliable confidence intervals (Bolker et al. 2012) | | | |
| ^c^ values between -1.96 and 1.96 indicate convergence; calculated using the *geweke.diag* function in the *coda* R package (Plummer et al. 2016) | | | |

# APPENDIX X: Degree of similarity to intraspecific aggressive signal model performance and pairwise comparisons from subset of data obtained from photographs

| **Table S23.** Performance of models explaining variation in the degree of similarity between in interspecific aggressive interactions and intraspecific aggressive interactions in subset of data obtained from photographs, after controlling for the effects of phylogeny using a Bayesian generalized linear mixed model approach (MCMCglmm). Models with lower DIC scores are considered to be better performing. | |
| --- | --- |
|  | |
| **model** | **DIC** |
| **Similarity between signals used in interspecific and intraspecific aggressive interactions** | |
| assumed ~ similarity | 626.09 |
| assumed ~ 1 (null) | 1246.00 |
|  | |

# APPENDIX XI: Signals used in aggressive interspecific interactions by family

| **Table S24a.** Variation in postures used in aggressive interactions by families with sufficient representation in our dataset. Values in the table are percentages of each family that use each position in a posture category in aggressive interspecific interactions. Boldface and gray shading indicate the most commonly used position(s) for each posture category in each family. | | | | | | | | | | | | | | | | | | | | |
| --- | --- | --- | --- | --- | --- | --- | --- | --- | --- | --- | --- | --- | --- | --- | --- | --- | --- | --- | --- | --- |
|  | **Body orientation** | | | | | | | **Head position** | | | | | **Wing position** | | | | | | | |
|  | forward.lowered | above | feet.forward | forward.normal | forward.upright | side.oriented | upside.down | forward.lowered | forward.upright | forward.normal | side.oriented | held.back.  upward | cosed.held.  slightly.out | closed.flat | closed.raised.  off.back | flapping | spread.outward | partially.spread | raised.upward | soaring.gliding |
| **Accipitridae** | **28%** | 6% | **28%** | 11% | 17% |  | 11% | **44%** | 22% | 17% | 6% | 11% | 6% |  |  | 17% |  | 17% | 11% | **28%** |
| **Anatidae** | **44%** |  |  | 22% | 22% | 11% |  | **56%** | 22% | 22% |  |  |  | **44%** | **44%** | 11% |  |  |  |  |
| **Ardeidae** | 14% |  |  | 21% | **64%** |  |  | 29% | **57%** | 14% |  |  | 29% | 14% |  | 7% | **43%** |  | 7% |  |
| **Columbidae** | 17% |  |  | 8% |  | **75%** |  | 8% |  | 33% | **58%** |  |  |  |  |  |  | 8% | **92%** |  |
| **Diomedeidae** | **40%** |  |  | **40%** | 10% | 10% |  | **60%** | 10% | 30% |  |  |  |  | 30% |  | **60%** | 10% |  |  |
| **Falconidae** |  | **50%** |  |  | 7% |  | 43% | **46%** | 8% | 23% | 23% |  |  |  |  | 43% |  |  | 7% | **50%** |
| **Fringillidae** | **50%** |  |  | 33% | 17% |  |  | **50%** | 25% | 25% |  |  | 8% | **33%** | 8% | 8% | 25% | 8% | 8% |  |
| **Icteridae** | 40% |  |  | 10% | **50%** |  |  | **60%** | 20% | 10% | 10% |  | **30%** |  | 10% | 20% | **30%** | 10% |  |  |
| **Psittacidae** | **40%** | 10% |  |  | 30% | 10% | 10% | **30%** | **30%** | **30%** | 10% |  | **40%** | 20% |  |  | 30% | 10% |  |  |
| **Spheniscidae** | **80%** |  |  |  | 20% |  |  | **70%** | 20% | 10% |  |  |  | **60%** |  |  | 10% | 20% | 10% |  |
| **Thraupidae** | **69%** |  |  | 11% | 19% | 1% |  | **61%** | 15% | 24% |  |  | **48%** | 35% | 6% | 3% | 5% | 3% | 1% |  |
| **Trochilidae** | 5% |  |  | 9% | **86%** |  |  | **50%** | 36% | 14% |  |  |  |  |  | **86%** | 5% |  | 9% |  |
| **Turdidae** | **75%** |  |  |  | 25% |  |  | **75%** | 17% | 8% |  |  | **50%** | 25% |  | 17% |  | 8% |  |  |
| **Tyrannidae** | 19% | 19% | 4% | 15% | **35%** | 4% | 4% | **54%** | 27% | 19% |  |  | 12% | 8% | 4% | **50%** | 12% | 12% | 4% |  |

| **Table S24b.** Variation in postures used in aggressive interactions by families with sufficient representation in our dataset. Values in the table are percentages of each family that use each position in a posture category in aggressive interspecific interactions. Boldface and gray shading indicate the most commonly used position(s) for each posture category in each family. | | | | | | | | | | | | | | | | | | | | | | |
| --- | --- | --- | --- | --- | --- | --- | --- | --- | --- | --- | --- | --- | --- | --- | --- | --- | --- | --- | --- | --- | --- | --- |
|  | **Bill position** | | | | | | | **Tail position** | | | | | | | | | | **Feet position** | | | | |
|  | open.forward | closed.forward | open.side | closed.side | closed.downward | open.downward | closed.upward | trailing.notfanned | trailing.fanned | down.fanned | down.notfanned | raised.fanned | partlyraised.  notfanned | partlyraised.  fanned | raised.notfanned | side.fanned | side.notfanned | on.substrate | extended | tucked.up | partly.extended | hanging |
| **Accipitridae** | 35% | **53%** | 6% |  | 6% |  |  | 36% | **57%** |  |  | 7% |  |  |  |  |  | **50%** | **50%** |  |  |  |
| **Anatidae** | **100%** |  |  |  |  |  |  | **100%** |  |  |  |  |  |  |  |  |  | **89%** |  |  |  | 11% |
| **Ardeidae** | **43%** | **43%** |  |  | 7% |  | 7% | **83%** | 17% |  |  |  |  |  |  |  |  | **100%** |  |  |  |  |
| **Columbidae** |  | 42% | 8% | **50%** |  |  |  | **50%** | 10% |  |  |  | 10% |  |  | 30% |  | **100%** |  |  |  |  |
| **Diomedeidae** | **89%** | 11% |  |  |  |  |  | **40%** |  |  |  | 10% | 20% | 10% | 20% |  |  | **100%** |  |  |  |  |
| **Falconidae** | 36% | **45%** |  | 18% |  |  |  |  | **77%** | 23% |  |  |  |  |  |  |  |  | **92%** |  | 8% |  |
| **Fringillidae** | **100%** |  |  |  |  |  |  | **75%** | 25% |  |  |  |  |  |  |  |  | **92%** |  |  | 8% |  |
| **Icteridae** | **60%** | 30% | 10% |  |  |  |  | 12% | **38%** | **38%** |  | 12% |  |  |  |  |  | **80%** | 10% |  | 10% |  |
| **Psittacidae** | **75%** | 12% | 12% |  |  |  |  | **78%** | 22% |  |  |  |  |  |  |  |  | **90%** |  |  | 10% |  |
| **Spheniscidae** | **100%** |  |  |  |  |  |  | **100%** |  |  |  |  |  |  |  |  |  | **100%** |  |  |  |  |
| **Thraupidae** | **95%** | 3% | 3% |  |  |  |  | **60%** | 18% | 4% | 8% | 3% | 5% |  |  |  | 1% | **98%** |  |  | 2% |  |
| **Trochilidae** | 17% | **83%** |  |  |  |  |  | 9% | 5% | **77%** | 9% |  |  |  |  |  |  | 13% | 25% | **56%** | 6% |  |
| **Turdidae** | **100%** |  |  |  |  |  |  | **63%** | 12% | 12% |  | 12% |  |  |  |  |  | **75%** | 17% |  |  | 8% |
| **Tyrannidae** | **83%** | 13% |  |  |  |  | 4% | **28%** | 24% | 24% | 12% |  | 4% | 4% |  | 4% |  | **55%** | 9% | 14% | 14% | 9% |

| **Table S24c.** Variation in postures used in aggressive interactions by families with sufficient representation in our dataset. Values in the table are percentages of each family that use each body region position in aggressive interspecific interactions. Boldface and gray shading indicate the most commonly used position(s) for each posture category in each family. | | | | | | | | | | | | | | | | | | | |
| --- | --- | --- | --- | --- | --- | --- | --- | --- | --- | --- | --- | --- | --- | --- | --- | --- | --- | --- | --- |
|  | **Shoulder position** | | | | | **Closest point** | | | | | | | | | | | | | |
|  | underwing.forward | wing.closed.  shoulder.visible | wing.horizontal | wng.closed.  shoulder.concealed | upperwing.forward | bill | bill.wing | bill.feet | bill.neck | bill.forehead | bill.face | bill.head | bill.throat.neck | bill.tail | feet | wing | breast | feet.tail | side |
| **Accipitridae** | **89%** | 6% | 6% |  |  | 44% |  |  |  |  |  | 6% |  |  | **50%** |  |  |  |  |
| **Anatidae** |  | 33% | 11% | **56%** |  | **100%** |  |  |  |  |  |  |  |  |  |  |  |  |  |
| **Ardeidae** | **50%** | 43% | 7% |  |  | **79%** |  |  | 14% |  |  |  | 7% |  |  |  |  |  |  |
| **Columbidae** | **50%** |  | 17% |  | 33% | 33% | 8% |  |  |  |  |  |  |  |  | **50%** |  |  | 8% |
| **Diomedeidae** | **60%** |  | 10% | 30% |  | **100%** |  |  |  |  |  |  |  |  |  |  |  |  |  |
| **Falconidae** | **100%** |  |  |  |  |  |  |  |  |  |  |  |  |  | **86%** | 7% |  | 7% |  |
| **Fringillidae** | **33%** | **33%** | 17% | 17% |  | **100%** |  |  |  |  |  |  |  |  |  |  |  |  |  |
| **Icteridae** | **50%** | 40% | 10% |  |  | **90%** |  |  |  |  |  |  |  |  | 10% |  |  |  |  |
| **Psittacidae** | 40% | **60%** |  |  |  | **90%** |  |  |  |  |  |  |  |  | 10% |  |  |  |  |
| **Spheniscidae** | 20% | **70%** | 10% |  |  | **90%** |  |  |  |  |  |  |  |  |  |  | 10% |  |  |
| **Thraupidae** | 5% | **79%** | 5% | 10% | 13% | **98%** |  | 1% |  |  | 1% |  |  |  |  |  |  |  |  |
| **Trochilidae** | **86%** |  | 14% |  |  | **100%** |  |  |  |  |  |  |  |  |  |  |  |  |  |
| **Turdidae** | 25% | **67%** |  | 8% |  | **92%** |  | 8% |  |  |  |  |  |  |  |  |  |  |  |
| **Tyrannidae** | **73%** | 23% |  |  | 4% | **85%** |  | 4% |  |  |  |  |  | 8% | 4% |  |  |  |  |

| **Table S25.** Variation in body regions highlighted in aggressive interactions by families with sufficient representation in our dataset. Values in the table are percentages of each family that highlight each body region in aggressive interspecific interactions. Boldface and gray shading indicate the most commonly highlighted body region(s) for each family. | | | | | | | | | | | | | | | | | |
| --- | --- | --- | --- | --- | --- | --- | --- | --- | --- | --- | --- | --- | --- | --- | --- | --- | --- |
|  | **Highlighted body regions** | | | | | | | | | | | | | | | | |
|  | mouth | bill | face.throat | breast | belly | sides | crown | nape.back | shoulders | upperwings | underwings | uppertail | uppertail.  coverts | undertail | undertail.  coverts | tarsal.  feathers | legs.feet |
| **Accipitridae** | 11% | 11% | 56% | 39% | 6% |  |  | 11% | 11% | 6% | **61%** | 6% |  |  |  |  | 28% |
| **Anatidae** | **100%** | 89% | 67% |  |  |  |  | 33% |  |  |  |  |  |  |  |  |  |
| **Ardeidae** |  | 14% | **64%** | 36% |  |  | 43% | 21% |  |  | 57% |  |  |  |  |  |  |
| **Columbidae** |  |  | 58% | 25% |  | 17% |  |  | 17% | 8% | **83%** |  |  |  |  |  |  |
| **Diomedeidae** | **70%** | **70%** | **70%** |  |  |  |  |  |  |  | **70%** |  |  |  |  |  |  |
| **Falconidae** |  |  | 29% |  | 7% |  |  |  |  |  | 50% |  |  | 43% |  |  | **79%** |
| **Fringillidae** | 42% | 17% | **75%** | 25% | 8% |  | 8% | 17% |  | 17% | 25% |  |  |  |  |  |  |
| **Icteridae** |  | 20% | **80%** | 20% | 10% |  | 10% |  | 20% |  | 30% | 20% |  |  |  |  | 10% |
| **Psittacidae** | 50% | 60% | **90%** | 10% | 10% |  |  |  |  |  | 30% |  |  |  |  |  | 10% |
| **Spheniscidae** | **90%** | 80% | 80% | 20% | 10% |  |  |  |  |  |  |  |  |  |  |  |  |
| **Thraupidae** | 64% | 46% | **91%** | 11% | 3% | 1% | 10% | 19% | 3% | 5% | 5% | 6% |  | 8% |  |  | 3% |
| **Trochilidae** | 9% | 23% | **91%** | 46% | 27% |  |  |  |  |  | 5% |  |  | 5% |  |  | 5% |
| **Turdidae** | **75%** | 33% | 67% | 8% |  |  |  | 50% |  |  | 17% | 8% |  | 8% |  |  | 17% |
| **Tyrannidae** | 58% | 12% | **62%** | 8% | 8% |  | 8% |  |  | 4% | 58% | 4% |  | 23% |  |  | 12% |

| **Table S26.** Variation in colors highlighted in aggressive interactions by families with sufficient representation in our dataset. Values in the table are percentages of each family that highlight each color in aggressive interspecific interactions. Boldface and gray shading indicate the most commonly highlighted color(s) for each family. | | | | | | | | |
| --- | --- | --- | --- | --- | --- | --- | --- | --- |
|  | Highlighted colors | | | | | | | |
|  | red/pink/  orange/yellow | blue/green/  violet | black | dark-white contrast | brown/beige | gray | white | rufous/  chestnut |
| **Accipitridae** | 44% |  | 22% | **72%** | 39% | 20% | 22% |  |
| **Anatidae** | **63%** |  | 33% | 11% | 56% |  | 11% | 44% |
| **Ardeidae** | 50% |  | 14% | 36% |  | 46% | **57%** | 50% |
| **Columbidae** |  |  | 42% |  | 58% | **83%** | 8% | 25% |
| **Diomedeidae** | 70% | N/A | 10% | 30% |  |  | **100%** | N/A |
| **Falconidae** | **71%** |  | 14% | 71% | 43% | 14% |  |  |
| **Fringillidae** | **83%** | 43% | 58% | 40% | 25% |  |  |  |
| **Icteridae** | 80% | 13% | **90%** |  |  |  |  |  |
| **Psittacidae** | **90%** | **90%** | 29% | 50% |  |  |  |  |
| **Spheniscidae** | 70% | N/A | **100%** |  |  |  |  | N/A |
| **Thraupidae** | 30% | 67% | 40% | 11% | 18% | 34% |  | 8% |
| **Trochilidae** | **86%** | 68% | 14% | 21% | 18% |  | 36% | 20% |
| **Turdidae** | **75%** | N/A | 57% |  | **75%** | 13% |  |  |
| **Tyrannidae** | 54% |  | 8% | **73%** | 39% | 12% | 8% |  |

# APPENDIX XII: Supporting Information References

Bolker, B., M. Brooks, B. Gardner, C. Lennert, and M. Minami. 2012. Owls example: a zeroinflated, generalized linear mixed model for count data. https://groups.nceas.ucsb.edu/non-linearmodeling/projects/owls/WRITEUP/owls.pdf.

Hadfield, J. D. 2010. MCMC methods for multi–response generalised linear mixed models: the MCMCglmm R package. Journalof Statistical Software 33:1–22.

Hadfield, J. D. 2018. MCMCglmm: MCMC generalised linear mixed models. R package version 2.26.

Hadfield, J. D. 2021. MCMCglmm Course Notes. https://cran.r-project.org/web/packages/MCMCglmm/vignettes/CourseNotes.pdf

Jetz, W., G. Thomas, J. Joy, K. Hartmann, and A. O. Mooers. 2012. The global diversity of birds in space and time. Nature 491: 444-448. https://doi.org/10.1038/nature11631

Plummer, M., N. Best, K. Cowles, K. Vines, D. Sarkar, D. Bates, R. Almond, and A. Magnusson. 2016. coda: output analysis and diagnostics for MCMC. R package version 0.19-1.
